# RAB27B controls palmitoylation-dependent NRAS trafficking and signaling in myeloid leukemia

**DOI:** 10.1172/JCI165510

**Published:** 2023-06-15

**Authors:** Jian-Gang Ren, Bowen Xing, Kaosheng Lv, Rachel A. O’Keefe, Mengfang Wu, Ruoxing Wang, Kaylyn M. Bauer, Arevik Ghazaryan, George M. Burslem, Jing Zhang, Ryan M. O’Connell, Vinodh Pillai, Elizabeth O. Hexner, Mark R. Philips, Wei Tong

**Affiliations:** 1 The State Key Laboratory Breeding Base of Basic Science of Stomatology (Hubei-MOST) and Key Laboratory of Oral Biomedicine, Ministry of Education, School and Hospital of Stomatology, Wuhan University, Wuhan, Hubei, China.; 2 Division of Hematology, Children’s Hospital of Philadelphia, Philadelphia, Pennsylvania, USA.; 3 Department of Pediatrics, Perelman School of Medicine at the University of Pennsylvania, Philadelphia, Pennsylvania, USA.; 4 Department of Biochemistry, School of Medicine at the Southern University of Science and Technology, Shenzhen, Guangdong, China.; 5 Department of Medicine and Perlmutter Cancer Center, New York University Grossman School of Medicine, New York, New York, USA.; 6 Department of Pathology, University of Utah, Salt Lake City, Utah, USA.; 7 Department of Biochemistry and Biophysics, Perelman School of Medicine, University of Pennsylvania, Philadelphia, Pennsylvania, USA.; 8 McArdle Laboratory for Cancer Research, University of Wisconsin–Madison, Madison, Wisconsin, USA.; 9 Pathology and Laboratory Medicine, Children’s Hospital of Philadelphia, Philadelphia, Pennsylvania, USA.; 10 Division of Hematology and Oncology, Abramson Cancer Center, University of Pennsylvania, Philadelphia, Pennsylvania, USA.

**Keywords:** Cell Biology, Hematology, Cancer, Signal transduction

## Abstract

RAS mutations are among the most prevalent oncogenic drivers in cancers. RAS proteins propagate signals only when associated with cellular membranes as a consequence of lipid modifications that impact their trafficking. Here, we discovered that RAB27B, a RAB family small GTPase, controlled NRAS palmitoylation and trafficking to the plasma membrane, a localization required for activation. Our proteomic studies revealed *RAB27B* upregulation in *CBL-* or *JAK2-*mutated myeloid malignancies, and its expression correlated with poor prognosis in acute myeloid leukemias (AMLs). *RAB27B* depletion inhibited the growth of *CBL*-deficient or *NRAS*-mutant cell lines. Strikingly, *Rab27b* deficiency in mice abrogated mutant but not WT NRAS–mediated progenitor cell growth, ERK signaling, and NRAS palmitoylation. Further, *Rab27b* deficiency significantly reduced myelomonocytic leukemia development in vivo. Mechanistically, RAB27B interacted with ZDHHC9, a palmitoyl acyltransferase that modifies NRAS. By regulating palmitoylation, RAB27B controlled c-RAF/MEK/ERK signaling and affected leukemia development. Importantly, *RAB27B* depletion in primary human AMLs inhibited oncogenic NRAS signaling and leukemic growth. We further revealed a significant correlation between *RAB27B* expression and sensitivity to MEK inhibitors in AMLs. Thus, our studies presented a link between RAB proteins and fundamental aspects of RAS posttranslational modification and trafficking, highlighting future therapeutic strategies for RAS-driven cancers.

## Graphical abstract

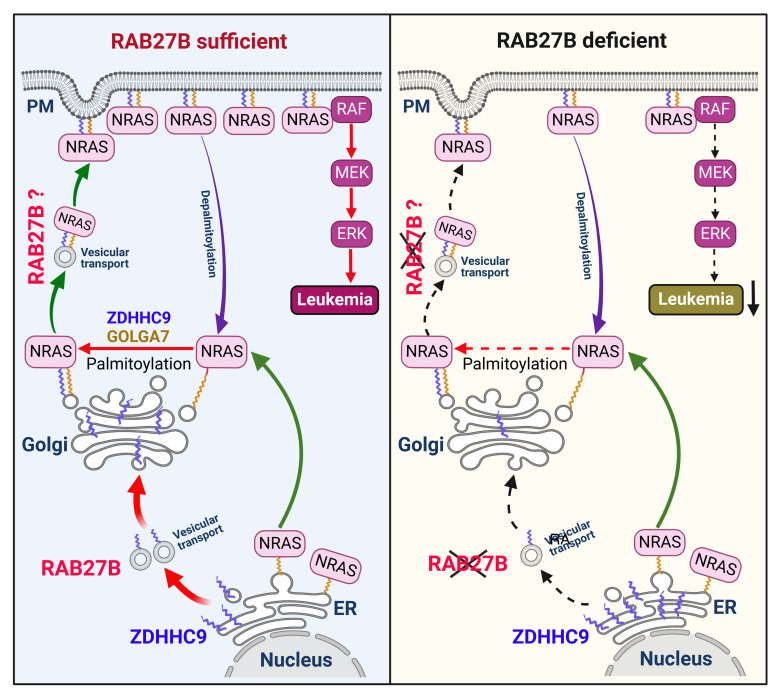

## Introduction

CBL family proteins, including C-CBL (also called CBL), CBL-B, and CBL-C, are RING domain–containing E3 ubiquitin ligases, among which CBL and CBL-B are widely expressed in hematopoietic cells. CBL family E3 ligases negatively regulate intracellular signaling of many critical receptor tyrosine kinases (RTKs) and cytokine receptors in hematopoietic cells, including FLT3 (1), c-kit (2), EPOR (3), and TPOR (4). CBL E3 ligases are known to regulate RTK internalization via nondegradative ubiquitination, independent of proteasome degradation (5). Deletions or loss-of-function mutations in *CBL* are found in a wide range of myeloid malignancies, in particular, myelodysplastic syndrome/myeloproliferative neoplasm (MDS/MPN) overlap syndromes (6, 7). MDS/MPN overlap syndromes include chronic or juvenile myelomonocytic leukemia (CMML and JMML), in which *CBL* mutations occur at a frequency of approximately 20% (7, 8). We previously reported that protein ubiquitination by CBL and CBL-B controls JAK2 stability and activity that is important for curbing hematopoietic stem and progenitor cell (HSPC) expansion and myeloid malignancies (9). JAK inhibitors are effective in treating CBL or JAK2 mutated myeloid malignancies, but they are not curative. Therapeutically relevant CBL targets remain poorly established.

RAS pathway mutations, including mutations in *RAS,* *CBL, NF1,* and *PTPN11* genes, are among the most prevalent mutations in solid and hematological malignancies (10, 11). The mammalian RAS proteins, HRAS, NRAS, and KRAS, belong to the small guanosine triphosphatase (GTPase) superfamily and serve as the master switches in growth factor receptor signaling. They cycle between active GTP-bound and inactive GDP-bound states. RAS plays critical roles in normal development, as well as in a number of human cancers. Mutations in the *KRAS* and *NRAS* genes are frequently identified, with mutations in NRAS more frequent than mutations in KRAS, in myeloid disorders (15%–60%), including acute myeloid leukemia (AML), atypical chronic myeloid leukemia (aCML), CMML, and JMML (12). *RAS* mutations cause hyperactivation of the RAF/MEK/ERK signaling pathway. A method to directly target RAS has eluded RAS biologists for decades, owing to the lack of druggable pockets on the surface of RAS proteins and picomolar affinity binding of RAS with GTP. Recent advances have resulted in the break-through development of targeted inhibitors (sotorasib and adagrasib) for one specific mutant form of KRAS (G12C), and these have recently been approved for the treatment of non-small cell lung cancer (13, 14). However, G12C mutations are rare in all solid tumors other than those in the lung and they rarely occur in leukemia.

One tenet of RAS biology is that signaling is dependent on the subcellular localization of the GTPase. Nascent RAS proteins are synthesized on free polysomes and encounter farnesyltransferase in the cytosol. After farnesylation of its C-terminal CaaX motif, they gain an affinity for the ER, where they encounter CaaX-processing enzymes. Following CaaX modification, most of the KRAS proteins directly proceed to the plasma membrane (PM). In contrast, NRAS and HRAS proceed to the Golgi apparatus, where they are palmitoylated by the ZDHHC9–GOLGA7 complex (DHHC domain-containing 9 palmitoyl- acyltransferase and Golgin subfamily A member 7, a Golgi complex-associated protein) (15). Palmitoylation increases the affinity of RAS proteins for membranes by up to 100-fold. This increased affinity creates a kinetic trap that enriches NRAS and HRAS at the Golgi membranes, allowing for subsequent trafficking on the cytoplasmic face of exocytic vesicles destined for the PM. At the PM, RAS encounters and can be activated by receptor-Grb2-SOS complexes. Activated RAS proteins recruit RAF to the PM where it becomes active and initiates the MEK/ERK signaling cascade. NRAS and HRAS are discharged from the membrane by depalmitoylation and move by retrograde transport back to the Golgi and/or ER for another round of palmitoylation. This dynamic cycle is important for RAS activation and function, because inhibition of either RAS palmitoylation or depalmitoylation abrogates RAS-mediated signaling or cell growth (16–18). Oncogenic RAS proteins transform cells only when associated with cellular membranes. Therefore, a mechanistic understanding of RAS lipid modification and trafficking may open up new avenues for better and more effective therapies in treating RAS-mutated myeloid malignancies.

RAB27A and RAB27B are small RAB GTPases that have been shown to function in the regulation of exocytic pathways, which involves intracellular vesicle trafficking, docking, and fusion with PM (19). Although RAB27A and RAB27B share similar functions, they also can be involved in different vesicles and different cell types, or can have distinct functions within the same cell type (20, 21). In addition, RAB27A and B control different steps of the exosome secretion pathway (20). In murine hematopoietic cells, Rab27b is highly expressed in megakaryocytes and is important for proplatelet formation (22, 23); it also influences neutrophil recruitment by regulating vesicle trafficking in neutrophils (24). RAB27B has been shown to be upregulated in solid cancers, which correlates with metastasis and poor survival (25). However, little is known about its role in hematopoietic malignancies or signaling via oncogenic pathways.

In this study, we identified that RAB27B promoted myeloid malignancies by regulating NRAS palmitoylation, trafficking, and signaling. RAB27B was upregulated by oncogenic CBL/JAK2/RAS signaling, implicating a potential link between *CBL* mutations, *JAK2^V617F^*, and RAS/ERK signaling. Notably, RAB27B was important for the growth of leukemia cells with *CBL* or *NRAS* mutations. Hence, we believe that this work uncovered a new signaling dynamic that enhances our understanding of compartmentalized RAS signaling, suggesting that targeting RAB27B may be therapeutically useful in oncogenic CBL/JAK2 and RAS driven myeloid malignancies.

## Results

### RAB27B is markedly upregulated in CBL/CBL-B–deficient and CBL-mutant cells.

**Figure 1:**
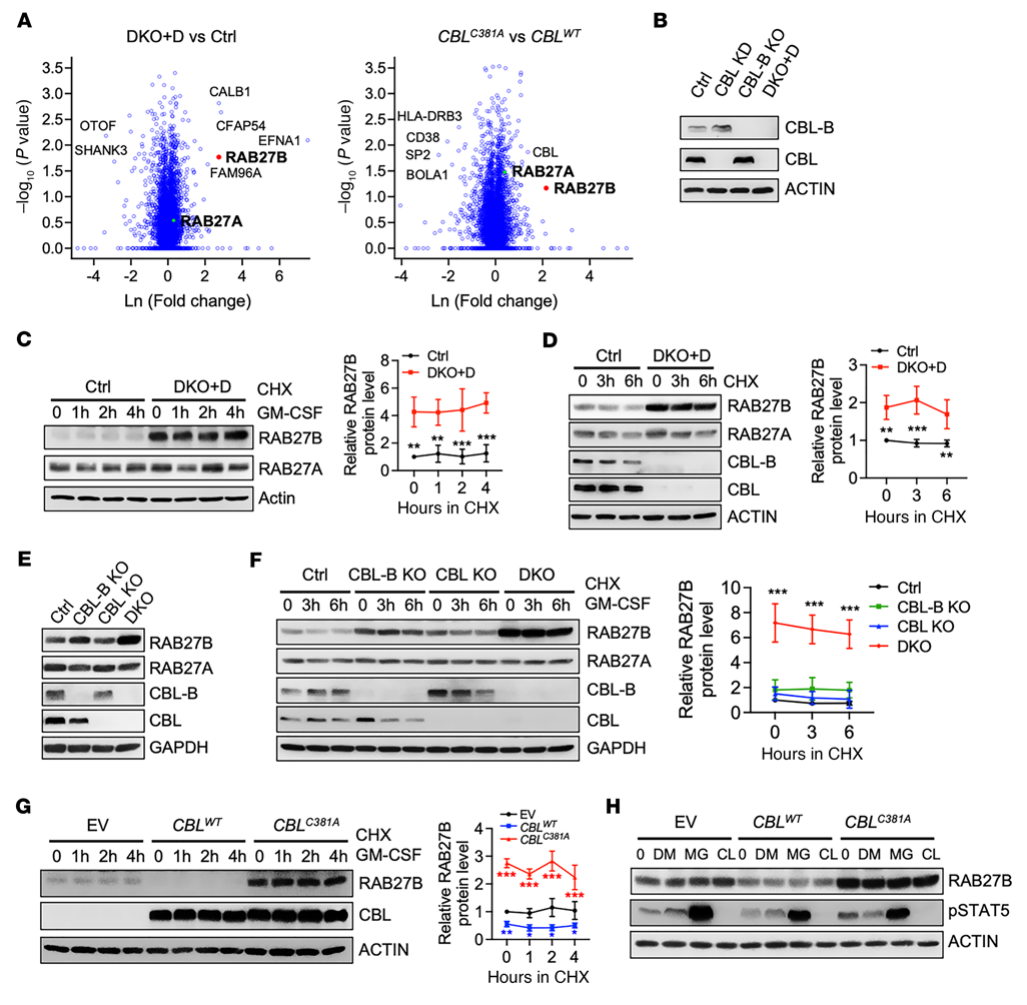
RAB27B is markedly upregulated upon *CBL* loss or inactivation. (**A**) Volcano plots comparing total protein levels between *CBL/CBL-B* double depleted (DKO+D) versus control (Ctrl) TF-1 cells, and between TF-1 cells overexpressing CBL^C381A^ E3-dead mutant versus CBL^WT^. Mean fold changes and *P* values were calculated from 3 independent quantitative proteomics experiments. (**B**–**D**) WB analysis to examine RAB27B and RAB27A protein levels and halflives (*n* = 3) in DKO+D compared with Ctrl cells, as shown in TF-1 (**B** and **C**) and U937 (**D**) cells. CHX: cycloheximide that inhibits nascent protein synthesis. Quantification of RAB27B half-lives is shown in the right panels of **C** and **D**. (**E** and **F**) WB analysis to examine RAB27B and RAB27A protein level and degradation (*n* = 3) in TF-1 cells after single or double knockout (DKO) of CBL and CBL-B. Quantification of RAB27B half-lives is shown in the right panel of **F**. (**G** and **H**) TF-1 cells stably expressing CBL^C381A^ mutant, CBL^WT^ or empty vector (EV) were established. (**G**) WB analysis to examine RAB27B protein halflife (*n* = 3). Quantification of RAB27B half-lives is shown in the right panel. (**H**) RAB27B protein level in the presence of DMSO, MG132 (MG, 10 μM) or Chloroquine (CL, 5 μM). pSTAT5 is used as a control for proteasomal degradation inhibited by MG132. Data for the halflife studies are represented as mean ± SD, and determined by 2-way ANOVA. *P < 0.05; **P < 0.01; ***P < 0.001.

To identify alterations in protein levels due to *CBL* loss or inactivation, we employed quantitative mass spectrometry (MS) to compare the whole-cell proteome of *CBL* knockdown/*CBL-B* knockout (DKO+D) with the control (Ctrl) and to compare cells overexpressing E3-dead C381A mutant with WT *CBL* in the erythroleukemia TF-1 cell line, as CBL/CBL-B-deficient and CBL-mutant cells were previously established (9) (Supplemental Table 1; supplemental material available online with this article; https://doi.org/10.1172/JCI165510DS1). Notably, we found that expression of the small GTPase RAB27B, but not its closely related family member RAB27A, was substantially increased in both *CBL/CBL-B*–null and *CBL*-mutant cells (Figure 1A). To confirm our MS results and determine if CBL regulated RAB27B protein stability, we performed cycloheximide (CHX) pulse-chase experiments to examine protein half-lives by Western blotting (WB). We found that RAB27B but not RAB27A protein levels were increased upon *CBL* loss in both TF-1 and U937 cells (Figure 1, B–D). However, the RAB27B halflife did not change in the time frame we examined. To further confirm our MS results, we constructed *CBL*/*CBL-B* single and double knockout (DKO) TF-1 cells using CRISPR/Cas9 technology. The results showed that *CBL/CBL-B* DKO dramatically increased RAB27B but not RAB27A protein levels compared with the Ctrl group and *CBL* or *CBL-B* single KO groups (Figure 1, E and F). Consistent with our MS results, RAB27B protein levels were markedly increased in TF-1–overexpressing C381A mutant *CBL*, while they were decreased in those expressing WT *CBL* compared with the empty vector (EV) group (Figure 1G). Interestingly, neither proteasome inhibitor MG132 nor lysosome inhibitor chloroquine changed the RAB27B protein level (Figure 1H). As a control, MG132 increased phosphorylated-STAT5 (pSTAT5), consistent with previous reports (26, 27). Taken together, our data revealed that *CBL/CBL-B* deficiency or inactivation modulated RAB27B protein level via its E3 ubiquitin ligase activity, but CBL did not appear to directly regulate the proteasomal degradation or stability of RAB27B.

### CBL/CBL-B deficiency or inactivation enhances RAB27B gene transcription.

**Figure 2:**
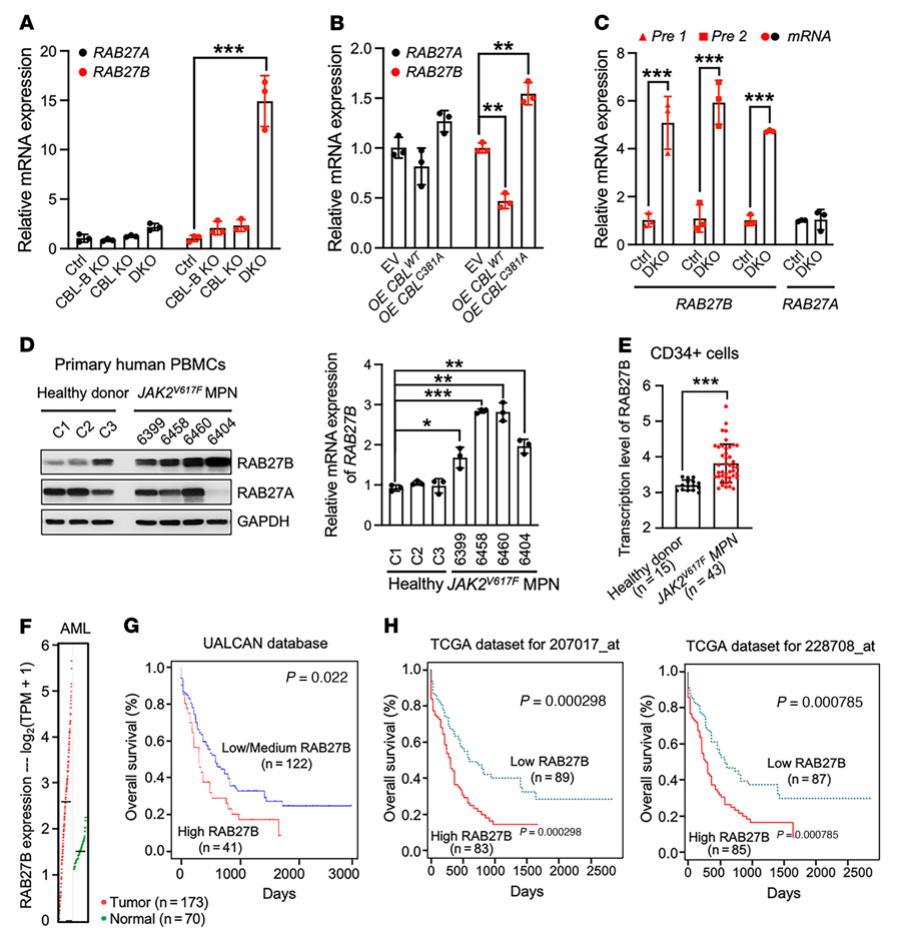
CBL depletion or inactivation enhances RAB27B gene transcription, and RAB27B expression correlates with poor survival in AML. (**A**) qRT-PCR to examine *RAB27B* and *RAB27A* mRNA levels in single or double knockout (DKO) of CBL and CBL-B compared with Ctrl TF-1 cells. (**B**) qRT-PCR to examine *RAB27B* and *RAB27A* mRNA levels in TF-1 cells stably expressing CBL^C381A^ mutant or CBL^WT^ compared with empty vector (EV). (**C**) qRT-PCR to examine *RAB27B* nascent and mature RNA level in TF-1 DKO cells compared with Ctrl cells. Two different pairs of primers were used to detect premRNA (designated as pre 1 and pre 2, depending on the primer set). Mature messenger RNA is labeled as mRNA. (**D**) RAB27B protein (left) and mRNA (right) levels in primary human PBMCs from healthy donors (C1–C3, *n* = 3) and patients with *JAK2^V617F+^* MPN (*n* = 4) are shown. (**E**) *RAB27B* mRNA levels in BM CD34^+^ cells from healthy donors (*n* = 15) and patients with *JAK2^V617F+^* MPN (*n* = 43) plotted using the expression data from GSE103176 (28). Each symbol indicates individual subject. (**F**) *RAB27B* expression level in patients with AML and healthy controls (GEPIA Cancer Database). (**G** and **H**) Kaplan-Meier plot of overall survival for patients with AML with low or high expression of *RAB27B*. UALCAN (**G**) top 25% or bottom 75% (low/medium) expression of RAB27B; CTGA database from BloodSpot (**H**) top 50% or bottom 50% expression of RAB27B. *P* values determined by log-rank *t* test are shown. In all relevant panels, data are represented as mean ± SD. 1-way ANOVA was used in panels **A**, **B** and **D**; Student’s 2-tailed *t* tests were used in Figure C and E; *P < 0.05; **P < 0.01; ***P < 0.001.

The observation that CBL and CBL-B do not control RAB27B protein stability prompted us to examine the *RAB27B* mRNA level. The mRNA level of *RAB27B*, but not *RAB27A* was markedly increased in TF-1 DKO cells compared with Ctrl or single KO cells (Figure 2A). *RAB27B* mRNA level was also increased in TF-1 cells overexpressing C381A mutant *CBL* and decreased in those expressing WT *CBL* compared with the EV group (Figure 2B). To assess if CBL regulates *RAB27B* transcription, we interrogated the precursor mRNA (premRNA) expression as opposed to *RAB27B* mature mRNA. PremRNA, also called primary transcript, is the first form of RNA synthesized in the nucleus before splicing, thus more faithfully representing the transcription rate. We found that *CBL/CBL-B* loss significantly upregulated *RAB27B* transcription in TF-1 cells, evidenced by the increased premRNA level (Figure 2C), suggesting that the CBL-controlled signaling network indirectly regulates *RAB27B* expression.

### Upregulated RAB27B expression is found in JAK2^V617F^ MPNs and correlates with poor survival in AMLs.

Since *CBL* loss enhances JAK2 activity and signaling (9), we examined *RAB27B* expression in primary cells from *JAK2^V617F^* MPN patients (Supplemental Table 2). We found that primary peripheral blood mononuclear cells (PBMCs) from *JAK2^V617F^* MPN patients showed significantly higher *RAB27B* mRNA and protein levels than healthy controls, but *RAB27A* expression was not significantly changed (Figure 2D). These data are consistent with a published genome-wide transcriptome analysis of CD34^+^ cells from a large number of MPN patients (28), demonstrating that CD34^+^ cells from *JAK2^V617F^* MPNs express a significantly higher level of *RAB27B*, but not *RAB27A*, compared with healthy controls (Figure 2E and Supplemental Figure 1A). To further study the correlation between JAK2 signaling and RAB27B expression, we compared multiple leukemia cell lines and found that they exhibit a wide range of RAB27B and RAB27A expression levels. The basal phosphorylation level of JAK2 was the highest in CMK cells, followed by *JAK2^V617F+^* HEL and SET-2 cells, and the lowest in K562 and TF-1 cells. Interestingly, RAB27B protein and mRNA levels seemed to be positively correlated with the basal JAK2 phosphorylation level in these cell lines (Supplemental Figure 1, B and C). We next sought to perturb JAK2 activation using various approaches to assess if JAK2 inactivation results in changes in RAB27B levels. Treatment for 24 hours, but not 5 hours, with the JAK2/1 inhibitor, ruxolitinib (Ruxo) in TF-1 CBL DKO cells significantly reduced *RAB27B* but not *RAB27A* mRNA levels (Supplemental Figure 1D). We stably expressed the myc-tagged WT JAK2 or the V617F mutant in TF-1 cells and found that *JAK2^V617F^* with aberrant JAK2 activation increased RAB27B, but not RAB27A, protein and mRNA levels (Supplemental Figure 1, E and F). As we previously reported, LNK acts as the adaptor protein for CBL-mediated JAK2 degradation, and *LNK*-deficient cells gain higher JAK2 protein level and enhanced JAK2 signaling (9). Consistently, the protein and mRNA levels of RAB27B, but not RAB27A, were increased in *LNK*-deficient cells (Supplemental Figure 1, G and H). In addition, we knocked down JAK2 in *JAK2^V617F^* HEL cells to approximately 50% and observed a corresponding reduction in RAB27B protein and mRNA levels (Supplemental Figure 1, I and J). Of note, TF-1 and HEL cells were not able to proliferate with sustained or complete KD of JAK2, as JAK2 is essential to their growth. Together, these data suggest that aberrant CBL signaling and JAK2 activation resulted in the upregulation of RAB27B transcription.

Furthermore, we analyzed *RAB27A* and *RAB27B* expression in AML using GEPIA Cancer Database (http://gepia.cancer-pku.cn/). We found that *RAB27B,* but not *RAB27A,* expression was higher in leukemia cells compared with normal cells (Figure 2F and Supplemental Figure 2A). Importantly, AML patients with high *RAB27B* expression show reduced survival compared with those with low *RAB27B* expression in 2 different data sets — UALCAN (http://ualcan.path.uab.edu/) (Figure 2G) and TCGA (https://servers.binf.ku.dk/bloodspot/) (Figure 2H) — consistent with a recent report (29). There was no significant correlation between *RAB27A* expression and AML patient survival (Supplemental Figure 2, B and C). These data reveal that oncogenic CBL/JAK2 signaling upregulated *RAB27B* and that *RAB27B* expression correlated with poor AML survival.

### RAB27B regulates NRAS activity, signaling, and leukemia cell growth.

**Figure 3:**
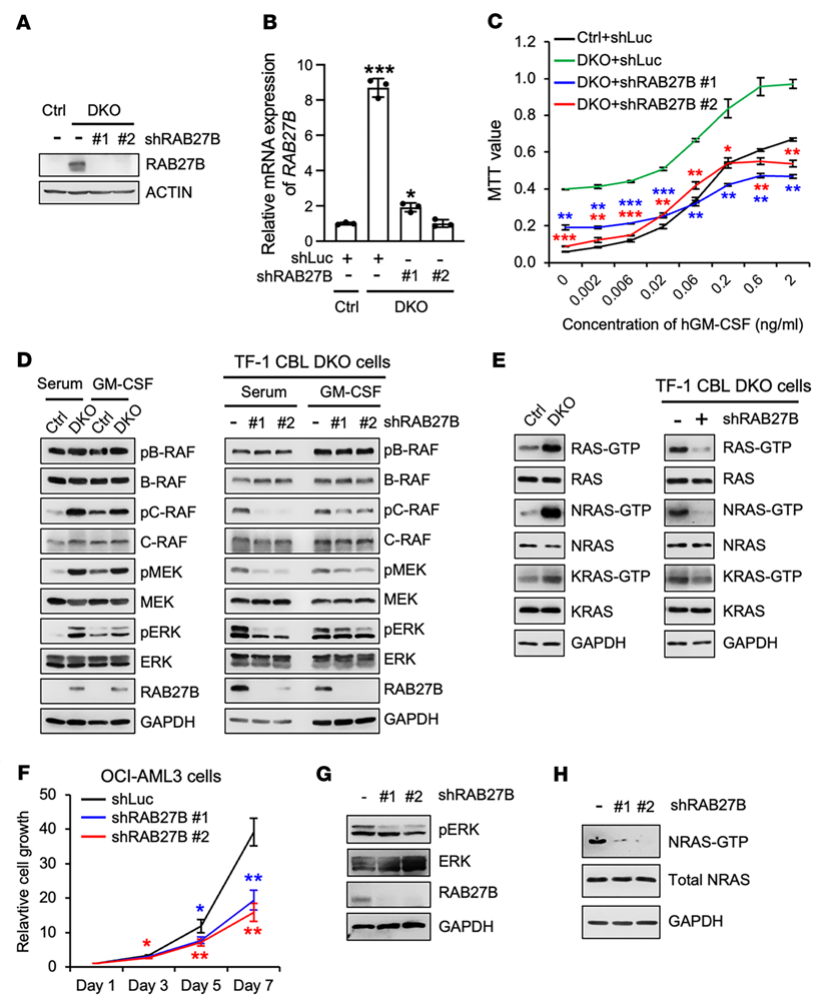
RAB27B regulates NRAS activity and signaling. (**A**–**E**) RAB27B was stably depleted via lentiviral-shRNA–mediated knockdown (KD) in TF-1 DKO cells, with shRNA against Luciferase (shLuc) used as a control. (**A** and **B**) KD efficiency of shRNA-RAB27B constructs was determined by WB (**A**) and qRT- PCR (**B**). (**C**) Cells were cultured in triplicates in different concentrations of human GM-CSF. Cell growth after 3 days in culture was determined by MTT absorbance. (**D**) TF-1 Ctrl or DKO cells (left), and TF-1 DKO cells with or without RAB27B depletion (right), were cultured in media containing serum only or serum and GM-CSF. Cell lysates were subjected to WB analysis with indicated antibodies to examine RAF/MEK/ERK activation. (**E**) TF-1 cells as described in (**D**) were cultured in media containing serum only. RAS GTPase activities were measured by RAS GTP pulldowns using RAF-1 RBD agarose beads, followed by WB with indicated antibodies. GTP-bound RAS represents active RAS. Input lysates were subjected to WB analysis with indicated antibodies as controls. (**F**–**H**) RAB27B was stably depleted via lentiviral-shRNA mediated KD in OCI-AML3 cells, with shLuc used as a control. (**F**) Cells were plated at equal cell numbers and cell growth was determined by counting of live cells. (**G**) ERK activation was determined by WB. (**H**) NRAS activity was determined by RAF-1 RBD agarose bead pulldown followed by WB using anti-NRAS antibodies. Input lysates were subjected to WB analysis with the indicated antibodies as controls. In all relevant panels, data are represented as mean ± SD, and 2-way ANOVA was used for comparing cell growth; *P < 0.05; **P < 0.01; ***P < 0.001.

The upregulation of RAB27B in *JAK2^V617F^* MPNs and its correlation with AML prognosis prompted us to investigate the potential role of RAB27B in myeloid malignancies. We first examined its function in the growth of TF-1 DKO cells, in which the RAB27B level is markedly elevated. We designed 2 efficient shRNAs targeting human *RAB27B* confirmed by WB and qRT-PCR (Figure 3, A and B). As we previously reported, TF-1 DKO cells exhibited cytokine-independent growth and were hypersensitive to cytokines compared with TF-1 parental cells (9). Notably, *RAB27B* depletion significantly blunted the growth of TF-1 DKO cells (Figure 3C), but not the growth of parental TF-1 cells (Supplemental Figure 3A). RAB27B is reported to be involved in exosomes and in cytokine secretion (30, 31). However, our data suggest that *RAB27B* depletion does not affect exosome secretion in TF-1–DKO cells (Supplemental Figure 4A). In addition, the growth of TF-1–DKO cells was not affected by the conditioned medium from TF-1-DKO cells and from RAB27B-depleted TF-1-DKO cells (Supplemental Figure 4, B–D), suggesting that RAB27B does not regulate TF-1 DKO cell growth via an autocrine pathway.

To study the mechanisms by which depletion of RAB27B inhibits leukemia cell growth, we evaluated intracellular signaling pathways important for the growth of hematopoietic cells, i.e., JAK-STAT, PI3K-AKT, and ERK. The basal AKT and ERK phosphorylation as well as JAK2 expression were elevated in TF-1 DKO cells compared with Ctrl cells, as previously reported (9) (Figure 3D and Supplemental Figure 5A). Importantly, we found a significant reduction in phosphorylated-ERK (pERK) upon *RAB27B* depletion in TF-1 DKO cells, but AKT or STAT5 phosphorylation remained unchanged (Figure 3D and Supplemental Figure 5B). In contrast, *RAB27A* depletion did not inhibit cell growth, and it did not affect the activation of AKT, ERK, and STAT5 signaling pathways in TF-1 DKO cells (Supplemental Figure 6, A–C). Of note, *RAB27B* depletion did not affect cytokine-induced signaling in TF-1 parental cells (Supplemental Figure 3B). We next assessed the upstream signals that activate ERK. Basal c-RAF and MEK phosphorylation were increased in TF-1 DKO cells compared with Ctrl cells (Figure 3D left panel). Importantly, *RAB27B* depletion dramatically reduced MEK and c-RAF phosphorylation in TF-1 DKO cells (Figure 3D right panel), implicating a potential involvement in RAS signaling. We thus performed RAS-GTP pulldowns using GST-RBD–conjugated beads, followed by WB analysis using isoform-specific RAS or pan-RAS antibodies. Total RAS activity, especially NRAS activity, and, to a lesser extent, KRAS activity, was elevated in TF-1 DKO cells compared with Ctrl cells (Figure 3E left panel). Strikingly, whereas activation of NRAS was almost completely abrogated upon *RAB27B* depletion in TF-1 DKO cells, GTP-loading of KRAS was preserved (Figure 3E right panel). We next validated our data in OCI-AML3 cells that harbor an *NRAS^Q61R^* mutation, a hotspot mutation that lies within the GTP-binding region of the NRAS protein. Consistently, *RAB27B* depletion reduced cell growth, ERK signaling, and NRAS activity in OCI-AML3 cells (Figure 3, F–H). Thus, our results revealed what we believe to be a previously unappreciated role for RAB27B in regulating NRAS activity, signaling, and leukemia cell growth.

### RAB27B is critical for PM localization and palmitoylation of NRAS.

**Figure 4:**
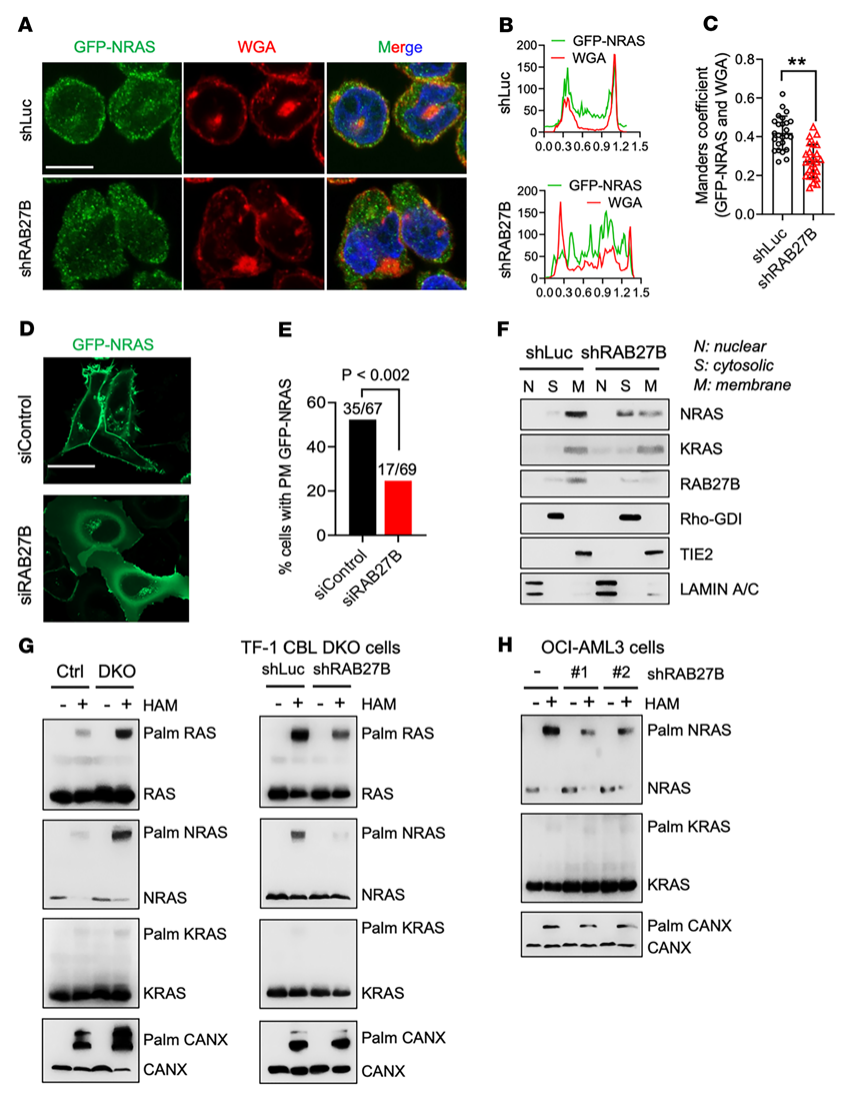
RAB27B is critical for NRAS plasma membrane localization and palmitoylation. (**A**–**C**) *RAB27B* silencing reduces NRAS localization in the PM in TF-1 DKO cells. TF-1 DKO cells with or without shRNA-mediated depletion of RAB27B were stably expressed GFP-NRAS via retroviral infection. (**A**) Representative immunofluorescent confocal images of GFP-NRAS (green) with the PM marker wheat germ agglutinin (WGA; red) and nucleus stain (DAPI; blue). Note that the intracellular WGA is endocytosed WGA. Scale bar: 10 μm. (**B**) A line was drawn across confocal images of cells as shown in **A**, and the signals for GFP-NRAS and WGA along the line are plotted. (**C**) Mander’s coefficient of GFP and WGA signals (mean ± SD), as shown in **B**. **P < 0.01, Student’s 2-tailed *t* test. (**D** and **E**) *RAB27B* silencing reduces NRAS localization in the PM in SK-MEL-147 cells. (**D**) Representative live-cell fluorescent images of GFP-NRAS in SK-MEL-147 cells transfected with siRNA to RAB27B (siRAB27B) or a nontargeting control (siControl). Scale bar: 20 μm. (**E**) Quantification of percentage of cells with PM–localized GFP-NRAS, as shown in **D**. Numbers over bars indicate number of cells with PM localization over the total number of GFP-NRAS cells. *P* < 0.002, determined by Fisher’s exact test. (**F**) TF-1 DKO cells with or without shRNA-mediated depletion of RAB27B were subjected to subcellular fractionation followed by WB analysis with the indicated antibodies. (**G**) TF-1 Ctrl or DKO cells (left), and TF-1 DKO cells with or without shRNA-mediated RAB27B depletion (right), were cultured in media supplemented with serum only. Palmitoylation status of endogenous RAS proteins was assessed using the APE assay. HAM, hydroxylamine; Palm, palmitoylated. (**H**) Palmitoylation status of endogenous RAS proteins in OCI-AML3 cells with or without RAB27B depletion was assessed using the APE assay.

It is well established that membrane association is required for RAS activation and signaling. At a steady state, pools of NRAS have been observed on the PM and the Golgi, as well as in the cytosol (32, 33). Since RAB27B is reported to be a Golgi-associated RAB GTPase that plays a role in vesicle transport (20, 34), we sought to examine if it plays a role in NRAS trafficking. PM association of GFP-NRAS stably expressed in TF-1 DKO cells via lentiviral transduction was diminished upon shRNA silencing of RAB27B (Figure 4, A–C). Because hematopoietic cells are small and difficult to image, we performed live-cell imaging of GFP-NRAS in SK-MEL-147 cells, a melanoma cell line that harbors an NRAS^Q61R^ mutation, the same NRAS mutation found in OCI-AML3. As we have previously reported (33), GFP-NRAS localized to the PM and Golgi in SK-MEL-147 cells. Silencing RAB27B with siRNA in these cells significantly diminished the PM but not the Golgi expression (Figure 4, D and E). We next evaluated the subcellular localization of endogenous NRAS with and without depletion of RAB27B in TF-1 DKO cells using detergent-free subcellular fractionation assays (33, 35). RhoGDI, TIE2, and LAMIN serve as markers for cytosol, membrane, and nuclear fractions, respectively. Though NRAS was recovered predominantly in the membrane fraction of control shRNA cells, it was distributed among both membrane and cytosolic fractions in *RAB27B*-deficient TF-1 DKO cells (Figure 4F and Supplemental Figure 6D), which is consistent with our imaging data. In contrast, *RAB27B* depletion did not affect the subcellular distribution of endogenous KRAS, and *RAB27A*-depletion did not affect NRAS localization (Figure 4F and Supplemental Figure 6D).

Palmitoylation of NRAS on cysteine 181 regulates its trafficking between the Golgi and PM, and, therefore, regulates its signaling (36). To detect RAS palmitoylation, we performed an acyl-PEG exchange (APE) assay that allows for the detection of endogenous protein palmitoylation in total cell lysates (37). We first set out to confirm previous findings and validated our APE assays by stably expressing WT and oncogenic NRAS proteins with and without their C181S mutant counterparts. The C181S mutation completely abrogated palmitoylation of WT and oncogenic NRAS, as well as basal activation of ERK in TF-1 cells (Supplemental Figure 7, A and B). Notably, disruption of NRAS palmitoylation and basal ERK phosphorylation abrogated cytokine-independent growth, whereas GM-CSF-mediated cell growth was unaffected (Supplemental Figure 7C). Using the APE assay, we found that the basal pan-RAS and NRAS palmitoylation levels were elevated in TF-1 DKO cell compared with Ctrl cells, while KRAS was not palmitoylated (Figure 4G left panel). More importantly, *RAB27B* depletion significantly inhibited pan-RAS, and, in particular, NRAS palmitoylation in TF-1 DKO cells (Figure 4G right panel). Consistently, *RAB27B* depletion also reduced NRAS palmitoylation in OCI-AML3 cells (Figure 4H). HRAS activity was very low in these cell lines, indeed, the effect of RAB27B on this RAS isoform could not be determined (data not shown). These data reveal that RAB27B is critical for NRAS palmitoylation and explain our observation that RAB27B is required for NRAS trafficking to the PM.

### Rab27b deficiency in mice inhibits oncogenic NRAS-mediated signaling, HSPC growth, and myeloid leukemia development in vivo.

**Figure 5:**
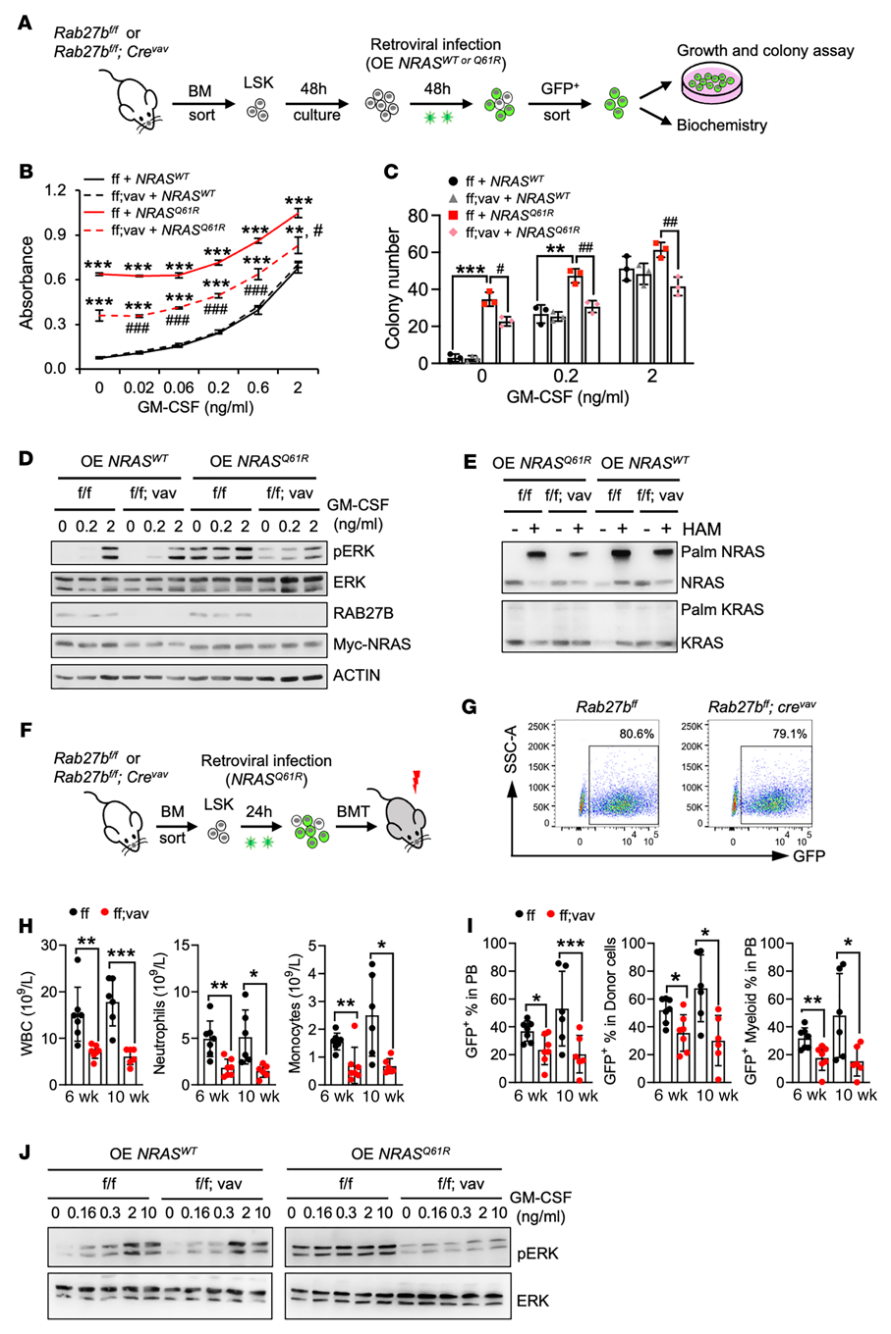
*Rab27b* deficiency in mice abrogates NRAS^Q61R^ -mediated signaling, cell growth, and myeloid leukemia development in vivo. (**A**–**E**) LSK cells from *Rab27b^fl/fl^* and *Rab27b^fl/fl^;Cre^vav^* mice were infected with retroviruses expressing either WT or Q61R-mutant NRAS, and, subsequently, GFP^+^ cells were purified by FACS. (**A**) Schematic illustration of experimental design. (**B**) Cells were cultured in triplicates in different concentrations of mouse GM-CSF and cell growth after 3 days in culture as determined by MTT absorbance is shown. (**C**) Infected HSPCs were plated in triplicates in a graded concentration of mouse GM-CSF. 7–10 days later, colony numbers were counted. (**B** and **C**) Data are represented as mean ± SD. *P* values are determined by 2-way ANOVA. *P < 0.05; **P < 0.01; ***P < 0.001; ^#^*P* < 0.05; ^##^*P* < 0.01; ^###^*P* < 0.001. For panel **B**, the asterisks indicate comparison to the ff+NRAS^WT^ group; the number signs indicate comparison to the ff+NRAS^Q61R^ group. (**D**) Infected HSPCs were stimulated with different doses of mouse GM-CSF and subjected to WB analysis. (**E**) Palmitoylation status of RAS was assessed using the APE assay. HAM, hydroxylamine; Palm, palmitoylated. (**F**–**J**) LSK cells from *Rab27b^fl/fl^* and *Rab27b^fl/fl^;Cre^vav^* mice were infected with retroviruses expressing Q61R mutant or WT NRAS, and subsequently transplanted into lethally irradiated recipient mice. (**F**) Schematic illustration of the *NRAS^Q61R^* transplant experimental scheme. (**G**) Flow cytometric plots showing the *NRAS^Q61R^* infection rates at the time of transplantation. (**H**) CBC analysis of recipient mice 6 and 10 weeks after transplantation. (**I**) Quantification of GFP^+^ donor CD45^+^ and myeloid cell percentages in the peripheral blood as well as GFP^+^ percentage in donor cells from each group at 6 and 10 weeks after transplantation. (**H** and **I**) Each symbol represents an individual mouse; bars indicate mean frequencies; error bars indicate SD. *P < 0.05; **P < 0.01; ***P < 0.001. 2-tailed *t* test. (**J**) Total BM cells from the transplanted mice were starved and stimulated with a graded dose of GM-CSF, and subsequently subjected to WB analysis.

To validate our findings in cell lines, we employed *Rab27b*-deficient mice. Germline *Rab27b* KO mice are grossly normal (38). Vav-cre expresses active Cre recombinase resulting in panhematopoietic deletion of the floxed sequences. To determine a cell-intrinsic role of RAB27B in hematopoietic cells, we generated conditional KO mice using Vav-cre (39). *Rab27b^fl/fl;Vav^* mice have largely normal hematopoiesis (data not shown). We isolated LSK (Lineage-Kit^+^Sca1^+^) cells from *Rab27b^fl/fl;Vav^* and *Rab27b^fl/fl^* mice and infected them with retrovirus expressing either WT or Q61R mutant NRAS with GFP as a fluorescent marker. Infected cells were purified and subjected to cell-based and biochemical studies (Figure 5A) (40). Oncogenic NRAS conferred cytokine-independent HSPC cell growth and colony-forming ability. This also occurred in the presence of low concentrations of GM-CSF (Figure 5, B and C). Strikingly, *Rab27b* deficiency significantly reduced mutant NRAS^Q61R^–conferred cell growth and clonogenic ability. In contrast, *Rab27b* deficiency had no effect on the growth of cells expressing NRAS^WT^ (Figure 5, B and C). Concordantly, *Rab27b* deficiency diminished ERK hyperactivation induced by the NRAS^Q61R^ mutation (Figure 5D) and reduced palmitoylation level of NRAS (Figure 5E). Hence, *Rab27b* deficiency in mice inhibits mutant NRAS-mediated signaling and cell growth.

To further study the requirement for RAB27B in mutant NRAS-mediated oncogenesis in vivo, we transplanted LSK cells expressing NRAS^Q61R^ into lethally irradiated recipient mice (Figure 5, F–J). *Rab27b^fl/fl^* and *Rab27b*-deficient cells had similarly high infection rates at the time of transplantation, as evidenced by the percentage of GFP^+^ cells (Figure 5G). At 6 and 10 weeks, mice transplanted with *Rab27b^fl/fl^* LSK cells expressing NRAS^Q61R^ exhibited high white blood cell counts, particularly neutrophils and monocytes (Figure 5H). In contrast, mice transplanted with *Rab27b-deficient* LSKs expressing NRAS^Q61R^ had significantly reduced white blood cell counts and lower proportions of GFP^+^ cells and GFP^+^ myeloid cells in the peripheral blood (PB) than those reconstituted with *Rab27b^fl/fl^* LSK cells expressing NRAS^Q61R^ (Figure 5, H and I). Importantly, mice transplanted with *Rab27b^fl/fl^* LSKs expressing NRAS^Q61R^ mostly developed MML, while those transplanted with *Rab27b-deficient* LSKs expressing NRAS^Q61R^ had reduced incidence of MML, instead the moribund mice died of T cell–acute lymphoblastic leukemia (T-ALL), as evidenced by analysis of the bone marrow and spleen (Table 1 and Supplemental Figure 8A). Moreover, BM cells from the mice transplanted with *Rab27b-deficient* LSKs expressing NRAS^Q61R^ showed reduced constitutive pERK activation compared with *Rab27b^fl/fl^* LSKs expressing NRAS^Q61R^ (Figure 5J), consistent with the ex vivo LSK data in Figure 5D.

**Figure 6:**
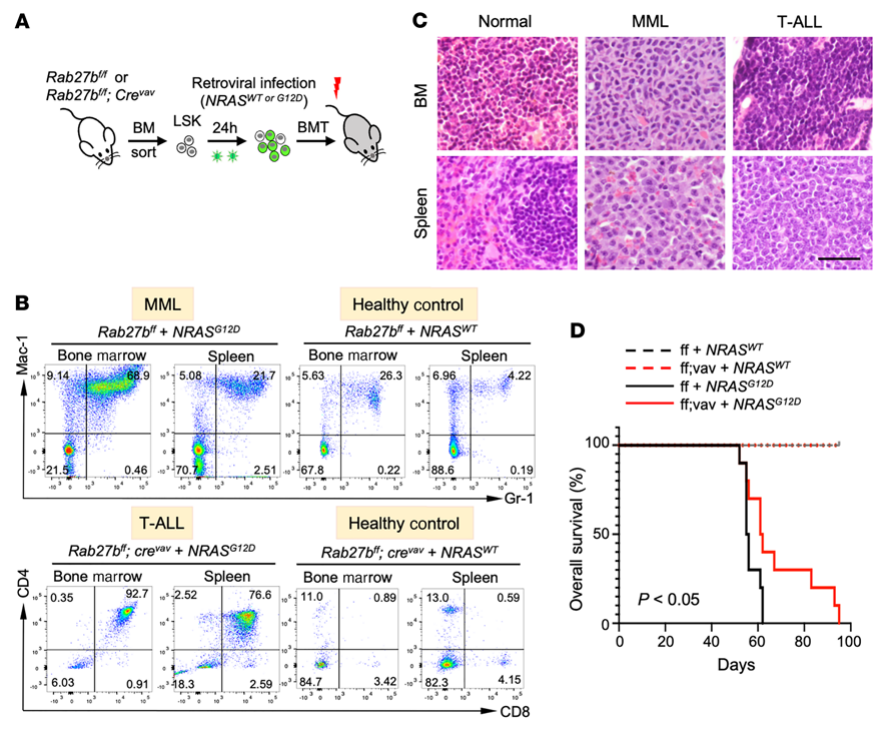
*Rab27b* deficiency in mice abrogates oncogenic NRAS^G12D^-mediated myeloid leukemia development in vivo. LSK cells from *Rab27b^fl/fl^* and *Rab27b^fl/fl^;Cre^vav^* mice were infected with retroviruses expressing G12D mutant or WT NRAS, and subsequently transplanted into lethally irradiated recipient mice. (**A**) Schematic illustration of the *NRAS^G12D^* transplant experimental scheme. (**B**) Representative flow cytometric plots of the bone marrow and spleen of the transplanted mice. GFP^+^ cells were gated for myeloid (Mac/Gr1) and T cell (CD4/CD8) lineages. (**C**) Representative histological analysis (H&E staining) of the bone and spleen of the transplanted mice are shown. Scale bar: 20 μm. (**D**) Kaplan–Meier survival curves of the transplanted mice. *P* value between ff+NRAS^G12D^ and ff;vav+Nras^G12D^ groups is calculated by log-rank *t* test.

To confirm our studies using mutant NRAS^Q61R^, we examined the effect of RAB27B in NRAS^G12D^, the most common mutant form of NRAS in human myeloid malignancies (41–43) (Figure 6). Consistent with our Q61R results, mice transplanted with *Rab27b^fl/fl^* LSKs expressing NRAS^G12D^ mostly developed MML, while those transplanted with *Rab27b-deficient* LSKs expressing NRAS^G12D^ had reduced incidence of CMML and instead died of T-ALL (Table 2), as evidenced by flow cytometric analysis (Figure 6B) and the histology of the bone marrow and spleen (Figure 6C and Supplemental Figure 8B). Consequently, mice transplanted with *Rab27b-deficient* LSKs expressing NRAS^G12D^ survived moderately but significantly longer than those transplanted with *Rab27b^fl/fl^* LSKs expressing NRAS^G12D^ (Figure 6D). Taken together, these data demonstrate that *Rab27b* deficiency abrogates oncogenic NRAS-mediated ERK signaling and myeloid leukemia development in vivo.

### RAB27B depletion reduces clonogenic growth, ERK activation, and NRAS palmitoylation in primary NRAS^mut^ AMLs.

**Figure 7:**
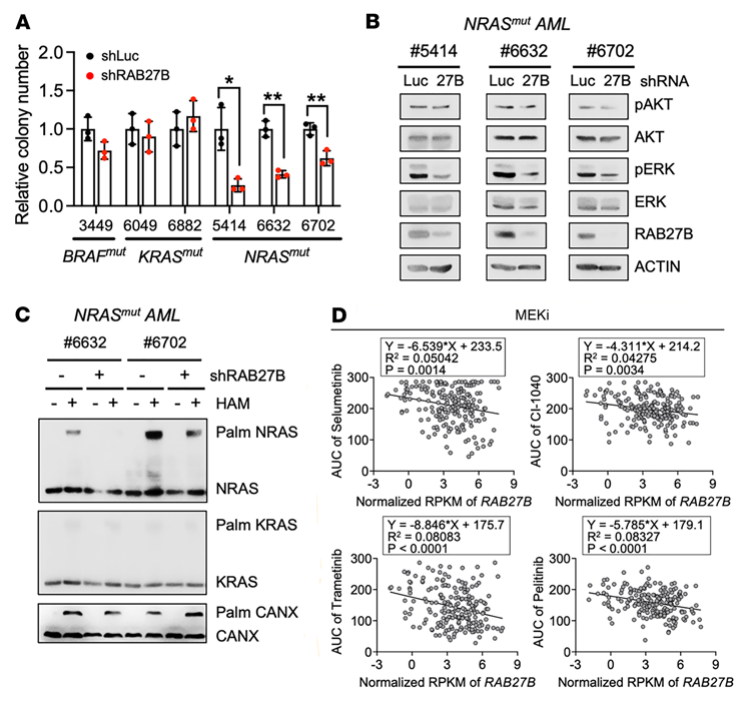
*RAB27B* depletion reduces clonogenic growth and NRAS/ERK signaling of primary *NRAS^mut^* AMLs. (**A**–**C**) Primary *NRAS^mut^* (*n* = 3) and *NRAS^WT^* (*n* = 3) AML cells were infected with lentiviruses expressing shRNA against luciferase (shLuc) or RAB27B (shRAB27B). Infected cells were purified by flow cytometric sorting and subjected to colony-forming assays and biochemical assays. (**A**) Relative colony numbers of primary human AMLs upon *RAB27B* depletion compared to that of shLuc controls. *P* values were determined by 2-tailed Student’s *t* test, *P < 0.05; **P < 0.01. (**B**) Primary human AMLs with or without *RAB27B* depletion were subjected to WB analysis with the indicated antibodies. (**C**) Palmitoylation status of endogenous RAS proteins was assessed using the APE assay. HAM, hydroxylamine. Palm: palmitoylated. (**D**) *RAB27B* expression levels in AMLs from patients in the BeatAML database are plotted with AUC to different MEK inhibitors. Linear regression trend line, *P* value and R^2^ value were generated using GraphPad Prism 8.0.

Our data suggest that RAB27B plays a critical role in the growth of NRAS mutant myeloid malignancies. To test the clinical significance of our findings, we examined primary *NRAS^mut^* or *NRAS^WT^* AML patient samples (Supplemental Table 3). We chose AMLs with high *NRAS^mut^* allele frequency in order to detect the full extent of *NRAS^mut^* effects in subsequent studies. We infected primary cells from the BM or PB of AML patients with lentiviruses expressing shRNA to *RAB27B* or luciferase (Luc) as a control. Of note, *RAB27B* depletion significantly reduced the colony formation ability of primary AML cells from *NRAS^mut^* patients, but not those of *BRAF^mut^* or *KRAS^mut^* patients (*NRAS^WT^*) (Figure 7A). These data are consistent with the signaling studies showing that *RAB27B* depletion reduced ERK phosphorylation in AMLs (Figure 7B). More importantly, *RAB27B* depletion reduced the palmitoylation level of endogenous NRAS proteins in primary AML cells (Figure 7C).

Our data suggest that RAB27B promotes AML cell growth by regulating NRAS/MEK/ERK signaling, therefore, we next interrogated data from BeatAML (44) to assess if *RAB27B* expression correlates with responses to MEK inhibitors (MEKi). Indeed, AML patients with high *RAB27B*, but not *RAB27A*, expression were sensitive to 4 different MEK inhibitors, as evidenced by lower AUC values (Figure 7D and Supplemental Figure 9A). However, no significant correlation was found between *RAB27B* expression and sensitivity to PI3K inhibitors (Supplemental Figure 9B). Thus, our data implicate a critical role for RAB27B in conferring aberrant NRAS/ERK signaling and AML cell growth.

### RAB27B interacts with ZDHHC9 and regulates ZDHHC9-mediated NRAS palmitoylation.

**Figure 8:**
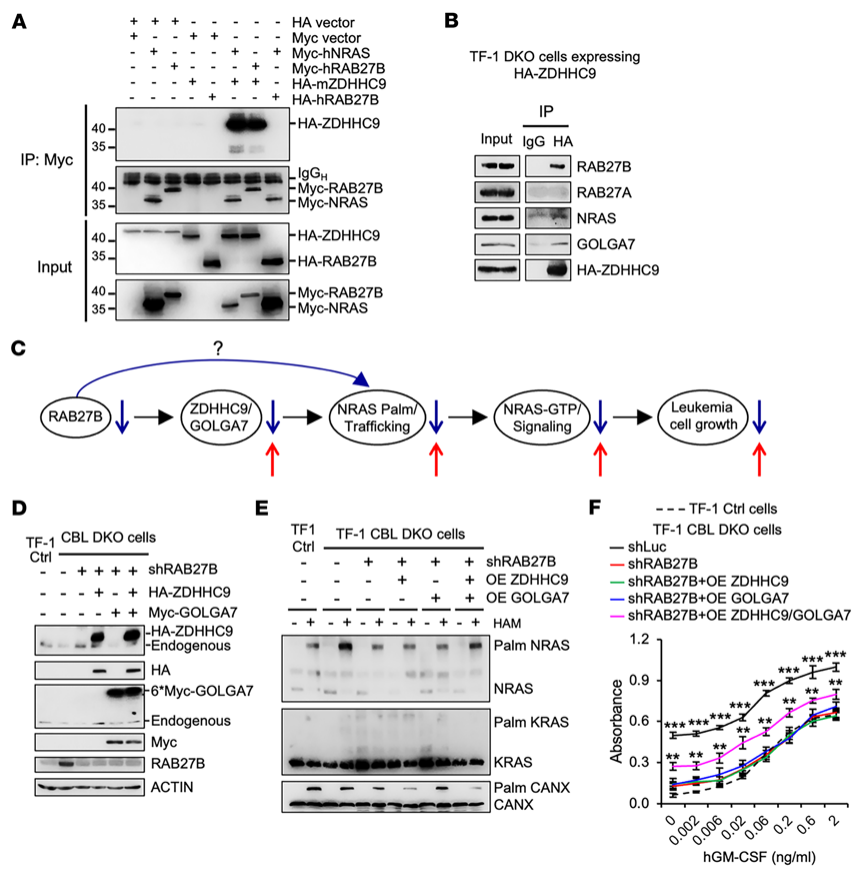
RAB27B binds to ZDHHC9 and regulates NRAS palmitoylation via ZDHHC9. (**A**) 293T cells were transfected with constructs to express tagged RAB27B, NRAS and ZDHHC9 as indicated. Cells were then subjected to IP followed by WB using the indicated antibodies. (**B**) TF-1 DKO cells stably expressing HA-ZDHHC9 were subjected to coIP with anti-HA antibodies followed by WB analysis using the indicated antibodies to examine its interaction with endogenous proteins. (**C**) Schematic illustration of the hypothesis depicting how RAB27B regulates NRAS signaling (blue arrows) and how to restore NRAS signaling disrupted due to RAB27B loss (red arrows). (**D**–**F**) RAB27B-depleted TF-1 DKO cells stably expressing HA-ZDHHC9 or Myc-GOLGA7 were subjected to examination of palmitoylation status and cell growth. (**D**) WB to examine the efficiency of RAB27B KD or ZDHHC9 and GOLGA7 overexpression in TF-1 Ctrl and DKO cells. (**E**) APE assay to examine palmitoylation of endogenous RAS proteins. OE, overexpression; HAM, hydroxylamine; Palm, palmitoylated. (**F**) Cells were cultured in triplicate in different concentrations of human GM-CSF and cell growth after 3 days in culture was determined by MTT absorbance. Data are represented as mean ± SD. **P < 0.01; ***P < 0.001 compared with the shRAB27B group, determined by 2-way ANOVA.

To explore the mechanisms by which RAB27B affects NRAS palmitoylation and trafficking, we first assessed the potential interaction between RAB27B, NRAS, and its palmitoyl acyltransferase (PAT) complex ZDHHC9/GOLGA7 by coimmunoprecipitation (coIP) (45). We overexpressed tagged RAB27B and NRAS in 293T cells and found that RAB27B pulled down ZDHHC9 but not NRAS (Figure 8A). Neither WT nor oncogenic mutant NRAS bound to RAB27B (Supplemental Figure 10A). RAB27B specifically interacted with ZDHHC9 but not GOLGA7 (Supplemental Figure 10B). In light of the recent discovery of ABHD17 as an acyl thioesterase that depalmitoylates NRAS (16), using coIP we examined whether RAB27B interacts with ABHD17. The results showed that RAB27B interacted with ZDHHC9 but not ABHD17A (Supplemental Figure 10, C and D). Importantly, we established TF-1 DKO cells stably expressing HA-tag ZDHHC9 and found that HA-ZDHHC9 pulled down endogenous RAB27B, but not RAB27A (Figure 8B). As expected, ZDHHC9 interacted with NRAS and GOLGA7 in 293T and TF-1 cells. Next, we overexpressed 23 HA-tagged mammalian ZDHHCs along with Myc-tagged RAB27B and found that ZDHHC9, 14, 18, and 23 bound to RAB27B. Among these ZDHHCs, ZDHHC9 and ZDHHC18 demonstrate the strongest interaction with RAB27B (Supplemental Figure 10E). However, ZDHHC18 depletion did not affect NRAS palmitoylation in TF-1 DKO cells (Supplemental Figure 11), consistent with previous reports that identified ZDHHC9 as a PAT that modifies NRAS (45).

Lastly, and importantly, we reasoned that if the reduced NRAS palmitoylation and the compromised cell growth observed in *RAB27B*-depleted cells was owing to disrupted ZDHHC9 function, overexpression of ZDHHC9 would be able to rescue this phenotype. To test this hypothesis, we first confirmed the role for ZDHHC9 in NRAS palmitoylation via shRNAs targeting ZDHHC9 or GOLGA7 (Supplemental Figure 12). In TF-1 CBL DKO cells, *ZDHHC9* depletion, and, to a lesser extent, *GOLGA7* depletion, reduced NRAS palmitoylation (Supplemental Figure 12C). Interestingly, RAB27B depletion exerted a stronger suppression of NRAS palmitoylation than depletion of ZDHHC9 did (Supplemental Figure 12C). Importantly, combined expression of ZDHHC9 and GOLGA7 partially rescued NRAS palmitoylation in *RAB27B*-depleted TF-1 DKO cells, while ZDHHC9 or GOLGA7 alone did not (Figure 8, C–E). Concordant with the palmitoylation data, dual expression of ZDHHC9 and GOLGA7 partially rescued the growth of *RAB27B*-depleted cells, but expression of ZDHHC9 or GOLGA7 alone did not (Figure 8F).

## Discussion

In this study, we uncovered a previously unappreciated role for RAB27B in regulating NRAS palmitoylation, subcellular trafficking, and signaling in myeloid malignancies, in part via interacting with the palmitoyl acyltransferase ZDHHC9. Furthermore, we identified a signaling axis, where oncogenic CBL/JAK2 signaling upregulates RAB27B to enhance NRAS activity and ERK phosphorylation, thereby implicating a potential link between *CBL* and *JAK2^V617F^* mutations and RAS/ERK signaling. Notably, RAB27B is important for the growth of leukemia cells with *CBL* or *NRAS* mutations but does not affect normal hematopoiesis. Hence, this work reveals what we believe to be new signaling dynamics that enhance our understanding of compartmentalized RAS signaling, suggesting RAB27B as a therapeutic target to abrogate oncogenic CBL/JAK2 and RAS-driven myeloid malignancies.

High *RAB27B* expression is shown to be an unfavorable prognostic factor in many solid cancers such as non-small cell lung carcinoma, colorectal cancer, and ovarian cancer (25); however, its role in leukemia was poorly established. In this study, we found that high *RAB27B* expression correlated with poor survival of patients with AML. The recent 2016 WHO classification of myeloid malignancies has called for the recognition of proliferative CMML (pCMML) and dysplastic (dCMML) subtypes, with the former having higher white blood cell counts (46). RAS pathway mutations, including *NRAS* and *CBL,* define the pCMML phenotype. This phenotype is aggressive, linked to dismal outcomes, and associated with increased transformation to AML, as demonstrated by the exome sequencing of primary human samples and the *Nras^G12D^* mouse model (47). Moreover, *RAS/CBL* mutations predict resistance to JAK inhibitors in myelofibrosis and are associated with poor prognostic features (48). The findings in this work provide a potential mechanistic link between CBL/JAK2–mutated myeloid malignancies and activation of RAS signaling via upregulation of RAB27B. Interestingly, the CBL family of E3 ubiquitin ligases and oncogenic JAK2 signaling does not directly control RAB27B protein stability; rather, they upregulate *RAB27B* gene transcription. Thus, our work suggests that the downstream gene network triggers a positive feedback loop to amplify oncogenic signaling and contribute to disease progression.

We showed in this study that *RAB27B* depletion dramatically reduced NRAS palmitoylation, activity, and downstream RAF/MEK/ERK signaling in both leukemia cell lines and primary human AMLs. *RAB27B* depletion impaired the PM localization of NRAS in both NRAS-mutated leukemia and melanoma cells. RAB27B interacted with ZDHHC9, a known PAT for NRAS, and regulated NRAS palmitoylation, thereby modulating NRAS trafficking to the PM. Importantly, overexpression of ZDHHC9 and GOLGA7 complex partially restored NRAS palmitoylation, signaling, and cell growth impacted by *RAB27B* depletion. The partial rescue suggests that RAB27B may affect the function of additional PATs for NRAS. This is corroborated by our data showing that *RAB27B* depletion exhibited a more pronounced effect on NRAS palmitoylation when compared with ZDHHC9 depletion or GOLGA7 depletion. We show that RAB27B interacted with ZDHHC9 but not NRAS or ABHD17, suggesting that RAB27B affected ZDHHC9 function. However, our present study does not address if and how RAB27B regulates ZDHHC9 activity or access to the substrate. How RAB27B functions and modulates NRAS palmitoylation cycle merits further future investigation.

Interestingly, whereas RAB27B is not required for the growth of parental TF-1 cells cultured in cytokines such as GM-CSF, it is critical for the growth of transformed cells cultured in basal conditions, specifically *CBL/CBL-B*–deficient TF-1 cells or *NRAS* mutated OCI-AML3 cells. In agreement, while *Rab27b^–/–^* mice display no obvious abnormalities, *Rab27b* deficiency significantly abrogated myeloid leukemia development conferred by oncogenic NRAS. Of note, *RAB27B* depletion impacted the palmitoylation and PM localization of both WT and oncogenic NRAS, but more effectively suppressed oncogenic NRAS-mediated leukemia cell growth and ERK signaling, suggesting that oncogenic NRAS–conferred cell growth is more sensitive to inhibition of palmitoylation or ERK signaling. This is consistent with prior studies showing that MEK inhibition is effective in abrogating murine Nras–mutant AML while Nras is dispensable for the normal function of HSCs (49). This can be attributed to the fact that KRAS plays an essential role in cytokine-mediated normal hematopoiesis (50). Furthermore, our mouse models utilizing retroviral-mediated overexpression of oncogenic NRAS suggest that RAB27B only impacts MML but not T-ALL development in mice. This might be due to the restricted expression pattern of Rab27b. Of note, this overexpression model infects both myeloid and lymphoid progenitors in addition to HSCs (51, 52), which limits its ability to dissect the potential distinct role of RAB27B in different progenitors. Nonetheless, our data are consistent with the notion that RAB27B plays a critical role in leukemia cell growth when NRAS is required — such as in AMLs with oncogenic *NRAS* or *CBL* mutations — while it is dispensable for the growth of AMLs with other dominant mutations or in normal HSPCs.

Considering that palmitoylation is a critical PTM for NRAS activation and signaling, significant efforts have been devoted to identifying specific PATs, depalmitoylases, and inhibitors to indirectly target RAS. It has been established that palmitoylation regulates the trafficking of NRAS between the Golgi and the PM (53), and palmitoylation of oncogenic *NRAS* is essential for leukemia progression (17). The dynamic palmitoylation/depalmitoylation cycle is important for RAS activation and function because inhibition of either RAS palmitoylation or depalmitoylation abrogates RAS-mediated signaling or cell growth (16–18). ZDHHC9 has been shown to palmitoylate H- and N-RAS in conjunction with GOLGA7 (45). However, previous studies and our data showed that depletion of ZDHHC9 only partially reduced NRAS palmitoylation and cannot completely abrogate leukemogenic potential of oncogenic NRAS (18, 54), thus suggesting that additional PATs can act on NRAS. Moreover, palmitoylation inhibitors cause cell damage due to their pleiotropic effects on lipid metabolism (55). Notably, depalmitoylation inhibitors disrupt the RAS palmitoylation cycle and its cellular localization, thus suppressing the growth of murine oncogenic *NRAS^mut^* AML blasts (36, 56). Recently, the ABHD17 family was found to be the relevant thioesterases that depalmitoylate NRAS (16). ABD957, an inhibitor of ABHD17, has been shown to be more selective than other ABHD inhibitors, though it only partially dampens oncogenic NRAS depalmitoylation and activity (16). Although we showed that RAB27B does not interact with ABHD17 directly in cell lines, we cannot exclude the possibility that RAB27B affects NRAS depalmitoylation.

Our findings indicate that RAB27B is a safe and promising therapeutic target for CBL or NRAS mutant malignancies. The RAB family are challenging targets for inhibition by small molecules, owing to the flat topology of RAB family effector interface and high affinity for GTP binding. Nexinhib-20 has been reported as a RAB27A inhibitor through inhibiting RAB27A-JFC1 binding (57). Cocrystal structures show that several effectors of RAB27A interact with the RAB27A SF4 pocket (WF-binding pocket) via a conserved tryptophan–phenylalanine (WF) dipeptide motif (58). A recent study took advantage of 2 cysteine residues, C123 and C188, that flank the WF pocket and are unique to RAB27A and RAB27B, which belong to a family of more than 60 RAB proteins, and identified the first covalent ligands for native RAB27A (57). This WF motif is present in RAB27B. Thus, this work provides a platform for identifying suitable lead fragments for future development of competitive inhibitors of the RAB27A and RAB27B-effector interaction interface. Alternatively, the WF pocket may allow the development of RAB27B degraders (59). Future investigations are warranted to discover specific inhibitors against RAB27B for the treatment myeloid malignancies.

## Methods

### Mice and primary human samples.

*Rab27b^fl/fl^* and *Rab27b^fl/fl^*; *cre^vav^* mice on the C57/BL6 background were generated as previously described (39). C57/B6.SJL mice were originally purchased from the Jackson Laboratories. Both male and female mice (8–12 weeks old) were used.

Primary BM– or PB– derived mononuclear cells from patients with MPN or AML were obtained from the Stem Cell and Xenograft Core Facility at the University of Pennsylvania. Patients’ primary cells were cultured in SFEM II media (STEMCELL) supplemented with 10% FBS, 10 ng/mL human IL-3, 50 ng/mL human stem cell factor (SCF) and 10ng/mL human GM-CSF at 37°C and 95% humidity in an atmosphere of 5% CO_2_. All cytokines were purchased from PeproTech.

### Cell lines.

OCI-AML3, U937, CMK, HEL, and K562 cells were cultured in RPMI media with 10% bovine calf serum (BCS). SET-2 cells were cultured in RPMI media with 20% BCS. TF-1 cells were maintained in RPMI supplemented with 10% BCS and 2 ng/mL human GM-CSF. 293T and SK-MEL-147 cells were cultured in DMEM with 10% BCS. TF-1, 293T, U937, HEL, and K562 cells were originally from ATCC. OCI-AML3 and CMK cells were originally from Deutsche Sammlung von Mikroorganismen und Zellkulturen (DSMZ). SK-MEL-147 melanoma cells were obtained from E. Hernando (New York University Langone Medical Center). All cell lines are grown at 37°C and 95% humidity in an atmosphere of 5% CO_2_.

### Constructs and antibodies.

MiR30-based shRNA constructs targeting *CBL, RAB27B, RAB27A*, *JAK2, LNK, ZDHHC18, ZDHHC9,* or *GOLGA7* were subcloned into lentiviral vectors (pCL20.MSCV.mir30.PGK.mCherry) provided by Shannon McKinney-Freeman at St. Jude Children’s Research Hospital (Memphis, Tennessee, USA). For CRISPR/Cas9-mediated knockout, human *CBL-B* gRNA were subcloned into a lentiGuideRFP vector. Human *CBL* gRNA was subcloned into an LRG vector provided by Junwei Shi (University of Pennsylvania). The shRNA sequences were: sh*CBL*: CCCGTACTATCTTGTCAAG; sh-human *RAB27B*: no. 1-CCGAATGGATCTTCAGGGAAAG, no. 2-AACAGAGCTTCTTAAATGTCAG; sh-mouse *Rab27b*: no. 1-ATGCCATGGGCTTCTTACTGAT, no. 2- CAGACATAGTATTAATTGGCAA; sh*RAB27A*: no. 1-AAGCTACGAAACCTCTCCT, no. 2-TAACTGATCCGTAGAGGCA; sh*JAK2*: TGGTTCAGGAGTTTGTAAA; sh*LNK*: CAAAGATGATGTCTGTCCG; sh*ZDHHC9*: no. 1- GTCTGTGATGGTGGTGAGAAAG, no. 2-AAGTCCTCATTTGCTTCTTTAC; shZDHHC18: GACGGAACTATCGCTTCTTCTA; sh*GOLGA7*: no. 1-AACAGTTCGAACTCTAAATAAC, no. 2-AACTCATTATGAGAAGGTTCTG. The gRNA sequences were: sg*CBL-B*: TGCCGCAGATCGCAGGACCG; and sg*CBL*: CAGCCCCCACCCGCCGGGGA.

The 23 HA-tagged ZDHHCs subcloned into the pEF-BOS vector were provided by Masaki Fukata (60). MSCV-Pgk-PAC-CBL WT and C381A mutant were gifted by E. Richard Stanley at Albert Einstein College of Medicine (New York, New York, USA). Mouse WT and V617F mutant JAK2 cDNAs were amplified by PCR from a homemade BaF3 cell cDNA library and constructed into a retroviral MigR1 (MSCV-ires-GFP)-Myc tag vector. Mouse ZDHHC9 was subcloned into the retroviral MigR1-Myc tag and retroviral pOZ-Flag-HA tag vectors. Human WT NRAS, RAB27B, RAB27A, and GOLGA7 cDNAs were amplified by PCR from a homemade TF-1 cell cDNA library and subcloned into a MigR1-Myc tag vector, pOZ-Flag-HA vector, or pEGFP vector (61). The lentiviral PCL20-GFP-tag vector was modified from the vector by Shannon McKinney-Freeman and used as previously described (61). G12D, Q61R, and C181S mutant NRAS constructs were generated from WT NRAS constructs using QuikChange mutagenesis.

Antibody details are as follows: anti-CBL antibody (no. 610441, 1:1,000) was from BC Biosciences; anti-RAB27B antibody (no. 18973, 1:700) was from Immuno-Biological Laboratories; anti-RAB27A antibody (no. ab55667, 1:1,000) was from Abcam; anti-Pan RAS antibody (no. STA-400, 1:1,000) was from Cell Biolabs; anti-KRAS (no. 12063-1-AP, 1:1,000) and HRAS (no. 18295-1-AP, 1:1,000) antibodies were from Proteintech; anti-ZDHHC9 antibody (no. MBS9128895, 1:1,000) was from MyBioSource; anti-GOLGA7 antibody (no. H00051125-M01, 1:500) was from Novus Biologicals; anti-CBL-B (no. sc-8006, 1:500), GAPDH (no. sc-365062, 1:2,000), ACTIN (no. sc-8432, 1:2,000), STAT5 (no. sc-74442, 1:2,000), BRAF (no. sc-5284, 1:1,000), CRAF (no. sc-133, 1:1,000), MEK (no. sc-81504, 1:1,000), NRAS (no. sc-31, 1:500), Rho GDI (no. sc-365190, 1:1,000), and LNK (no. sc-393709, 1:500) antibodies were from Santa Cruz Biotechnology; anti-pY1007/1008-JAK2 (no. 3776, 1:1,000), JAK2 (no. 3230, 1:1,000), pY697-STAT5 (no. 9351, 1:1,000), pSer445-BRAF (no. 2696, 1:1,000), pSer3388-CRAF (no. 9427, 1:1,000), pSer217/221-MEK (no. 9154, 1:1,000), pT202/204-ERK1/2 (no. 4370, 1:3,000), ERK1/2 (no. 9102, 1:1,000), pS473-AKT (no. 4060, 1:1,000), AKT (no. 9272, 1:1,000), CALNEXIN (no. 2679, 1:1,000), TIE2 (no. 4224, 1:1,000), MYC-Tag (no. 2276 and no. 2278, 1:1,000), HA-Tag (no. 2367 and no. 3724, 1:1,000) antibodies were from Cell Signaling Technology; Conjugated anti APC-c-Kit (no. 105812, 1:200), PE-Sca1 (no. 108108, 1:200), PE-Cy7-CD45.1 (no. 110730, 1:200), APC-Cy7-CD45.2 (no. 109824, 1:200), PE-B220 (no. 103208, 1:200), APC-CD3 (no. 100236, 1:200), PE-Gr1 (no. 108408, 1:200), and APC-Mac1 (no. 101212, 1:200) antibodies were from BioLegend.

### Quantitative proteomics, protein half-life and WB assays.

Stable isotope labeling of amino acids in cells (SILAC) MS was used for quantitative whole cell proteomics. Briefly, TF-1 CBL DKO cells or TF1 cells overexpressing *CBL C381A* mutant were cultured in RPMI media for SILAC (Thermo Fisher Scientific) containing dialyzed fetal bovine serum, nonessential amino acids (Thermo Fisher Scientific), and ^13^C-Lysine (K8) and ^13^C-Arginine (R10) (Cambridge Isotope Laboratories) along with standard supplements for 2 weeks. Cells were washed twice in cold PBS, and cell pellets were collected by centrifugation at 16,000*g* for 15 seconds at 4°C, then snap frozen at –80°C. Three biological replicates were subjected to quantitative proteomics analysis at the Children’s Hospital of Philadelphia Proteomics Core Facility.

For protein half-life assays, the cells were treated with 50 μg/ml cycloheximide (CHX) for the indicated time. The cell pellets were collected as described above, lysed in SDS lysis buffer and then boiled for 10 minutes at 95^o^C. For WB assay, 20 μg of each protein sample was loaded in an SDS-PAGE gel and then transferred to a nitrocellulose membrane (Bio-Rad) using an Amersham TE 70 ECL Semi-Dry Transfer Unit. The membranes were blocked for 30 minutes using 5% BSA (for phosphoprotein antibodies) or 5% nonfat milk (for total protein antibodies) in TBS + Tween20 (TBST) and incubated with primary antibodies using the same buffer at 4°C overnight, followed by incubation with HRP-conjugated secondary antibodies for 45 minutes at room temperature (RT). The membranes were subsequently washed in TBST and developed with ECL using a KwikQuant imager (Kindle Biosciences, LLC).

### IP.

Cells were lysed with IP buffer (10 mM Tris pH7.4 (Sigma-Aldrich), 150 mM NaCl (Sigma-Aldrich), 1% NP-40 (USB Corporation), Proteinase inhibitor cocktail (Roche), 1 mM NaF, 1 mM Na_3_VO_4_ (Sigma-Aldrich) and PMSF (Sigma-Aldrich) in deionized H_2_O) for 30 minutes at 4°C. The supernatant was collected by centrifugation at 16,000*g* for 5 minutes at 4°C. In some experiments, cell extracts were cleared by a 200,000*g* ultracentrifugation. After preclearing with protein A/G beads for 1 hour, cell lysates were incubated with HA-EZ Agarose Beads (Sigma-Aldrich) for 3 hours or anti-Myc primary antibody for 3 hours followed by incubation with protein A/G beads for 1 hour at 4°C with gentle agitation. The beads were then extensively washed with IP buffer and boiled for 10 minutes at 95^o^C in SDS loading buffer for WB analysis.

### RAS GTPase assay.

RAS activity was detected using the Ras Activation Assay kits from Cell Biolabs. Briefly, cell pellets were lysed with 1 × Assay Buffer supplemented with PMSF and proteinase inhibitor cocktail by pipetting up and down. The supernatant was collected by centrifugation at 16,000*g* for 10 minutes at 4°C. Next, 40 μL resuspended RAF1 RBD Agarose Beads were added to 1 mL cell lysate and incubated in cold room with gentle agitation. After an 1 hour incubation, the beads were pelleted by centrifugation at 16,000*g* for 15 seconds at 4°C, extensively washed with 1 × Assay Buffer and boiled for 10 minutes at 95°C in SDS loading buffer for WB analysis.

### Cell fractionation assay.

6 × 10^7^ TF-1 cells were resuspended in 2 mL hypotonic homogenization buffer (10 mM HEPES at pH 7.4, 10 mM KCl and 1.5 mM MgCl_2_), and kept on ice for 10 minutes. The swelled cells were dounced approximately 20 times using a dounce homogenizer with a tightly fitting pestle and cell lysis was confirmed under microscopy. The nuclei were collected by centrifugation at 500*g* for 10 minutes at 4^o^C and sonicated in 3mL 1 × SDS loading buffer, then boiled for 10 minutes at 95^o^C. Postnuclear supernatant was transferred to a polycarbonate tube and subjected to ultracentrifugation at 350,000*g* for 1 hour at 4°C. Supernatant was collected as the cytosolic fraction, and the pellet was lysed in 2 mL 1% NP-40 lysis buffer for 30 minutes at 4°C as the membrane fraction. 1 mL 3 × SDS loading buffer was added to the cytosolic and membrane fractions, which were then boiled for 10 minutes at 95°C. All fractions were then subjected to WB analysis.

### APE assay.

Protein palmitoylation was examined using the APE assay (37). In brief, cells were lysed in 1 × TEA lysis buffer (50 mM triethanolamine pH 7.3, 150 mM NaCl) supplemented with 5 mM EDTA, 4% SDS, and protease inhibitors cocktail in deionized H_2_O and directly sonicated at RT. The cell lysate was reacted with Tris (2-carboxyethyl) phosphine (TCEP; Pierce) for 30 minutes at RT with nutation and subsequently with N-ethylmaleimide (NEM; Sigma-Aldrich) for 2 hours to reduce and block free cysteine residues. Prechilled methanol, chloroform, and deionized H_2_O (4:1.5:3) were sequentially added to remove NEM and precipitate proteins, and this step was performed 3 times. Proteins were dissolved in 1 × TEA lysis buffer supplemented with 5 mM EDTA and 4% SDS and split into 2 tubes treated with or without 1 M hydroxylamine (HAM; Sigma-Aldrich) in 0.2% Triton X-100/TEA buffer for 1 hour at RT with nutation to remove S-fatty acid groups, followed by another 3 repeats of methanol-chloroform-H_2_O precipitation. The exposed cysteines were incubated with 1.33 mM 10 kDa mPEG-Mal (Sigma-Aldrich) for 2 hours at RT. Subsequently, proteins were subjected to methanol-chloroform-H_2_O precipitation once and boiled in SDS loading buffer for WB analysis.

### Real-time quantitative PCR.

Total RNA was isolated using the RNeasy Plus Mini Kit (#74134, QIAGEN). Reverse-transcription reactions were performed to synthesize cDNA using qScript cDNA Supermix (#95047, Quanta Biosciences). Real-time quantitative PCR (qRT-PCR) were performed using SYBR Green Master Mix (Applied Biosystems) in a ViiA 7 real-time PCR system (Applied Biosystems). The qRT-PCR primers used are as follows: RAB27B-preRNA-F: no. 1-CTGGAATAAGAGCAGTCATTTGACATC, no. 2-ACTGGGAAGAGGAAGTAACTTGGCCA; mRNA-F: TGCGGGACAAGAGCGGTTCCG; RAB27B-R: GCCAGTTCCCGAGCTTGCCGTT; RAB27A-F: GAAGCCATAGCACTCGCAG AG, RAB27A-R: ATGACCATTTGATCGCACCA; ZDHHC9-F: GAAGGTGACACGGAA ATGGG, ZDHHC9-R: GGCAGCAAATACAGGGATGG; ZDHHC18-F: GCAAGCTGACCC TTGCCATC, ZDHHC18-R: GGATCCCAGGGTCGGTGAAG; GOLGA7-F: ATGCAGAAGC AGAGAAGCTC, GOLGA7-R: GAGGCCTTGTGGAGCATAGA; GAPDH-F: CCACCCATGG CAAATTCC, and GAPDH-R: TGGGATTTCCATTGATGACAAG.

### Purification and quantification of exosomes.

2 × 10^6^ cells were cultured in 6 mL RPMI plus 10% exosome-depleted serum (Gibco). 1 mL of the media was collected after 1 hour, 3 hours, and 5 hours of culture. For purification of exosomes, the collected media was differentially centrifuged at 2,000*g* for 10 minutes then at 16,000*g* for 1 hour at 4°C to remove cells, debris, and bigger vesicles. Subsequently, the supernatant was loaded with a 1 mL syringe into a NanoSight NS300 (Malvern Instruments) for quantification of exosomes.

### Immunofluorescence and live-cell imaging.

TF-1 DKO cells were infected with pCL20-GFP-NRAS along with retroviruses expressing shLuc or shRAB27B. After 2 days, cells were incubated with Alexa Fluor 647–conjugated wheat germ agglutinin (WGA) for 10 minutes at 37^o^C to stain the PM. Subsequently, the cells were washed with cold PBS, fixed in 4% PFA for 10 minutes and resuspended in PBS with DAPI for imaging using a Zeiss LSM 710 confocal microscope equipped with a 100 × /1.4 numerical aperture oil-immersion objective. SK-MEL-147 cells were grown on 35 mm dishes with no. 0 coverslips (MatTek) and transfected with 25 nM ON-TARGETplus Human RAB27B siRNA SMARTPool (Dharmacon, Life Technologies) or nontargeting Control Pool siRNA (Dharmacon, Life Technologies) using DharmaFECT1 (Dharmacon, Life Technologies). Two days after siRNA transfection, the cells were transfected with 1 μg/dish pEGFP-NRAS using Lipofectamine 3000 Transfection Reagent (Thermo Fisher Scientific) according to the manufacturer’s instructions. The next day, EGFP fluorescence in live cells was imaged using a Zeiss LSM 800 inverted confocal microscope equipped with a 63 × /1.4 oil objective. Images were analyzed in ImageJ software.

### Lentiviral infection of primary human AML cells.

As described previously (62), non-TC-treated plates were precoated with RetroNectin (Takara) at 4°C overnight, followed by blocking with 2% BSA in PBS for 30 minutes at RT and washing with HBSS (Gibco). The lentiviral supernatant expressing shRNA targeting *Luc* or *RAB27B* was loaded to the plates and centrifuged at 1,800*g* for 1 hour at 10°C. After the plate was warmed to RT, viral supernatant was removed and human primary cells at 500,000 cells/mL with 2 μL/mL LentiBlast Premium (OZ Biosciences) were added to the plate, then centrifuged at 450*g* for 90 minutes at 37°C.

### Purification and retroviral infection of mouse LSK cells.

LSK cells were purified as described previously (61). In brief, BM cells were isolated from femora and tibiae of mice in PBS containing 0.5% BSA and 2 mM EDTA buffer. Lineage negative cells (Lin-) were isolated using the Lineage Cell Depletion Kit (Miltenyi Biotec). Lin- cells were stained with APC-c-Kit and PE-Sca1 antibodies, and LSK cells were purified with a BD FACSAria Fusion cytometer and cultured in SFEM medium plus 10% FBS supplemented with 20 ng/mL Flt3L, 20 ng/mL IL-6, 100 ng/mL SCF, 20 ng/mL TPO, and 0.1 mM β-ME. After 48 hours of culture, LSK cells were spin-infected with retroviruses expressing *WT* or *Q61R* mutant *NRAS* preloaded on RetroNectin- coated plates, with 10 μg/ml polybrene (Sigma-Aldrich). Cells were either transplanted 24 hours after infection or sorted for GFP positivity 48 hours after infection for cell proliferation assay, colony assay, or biochemical experiments.

### Signaling studies.

For cytokine sensitivity, LSK cells were starved in RPMI-1640 media plus 0.5%BSA for 1–2 hours, then stimulated with a graded dose of GM-CSF for 10 minutes. Cell pellets were immediately collected by centrifugation at 16,000*g* for 15 seconds and snapfrozen. For TF-1 cells, cells were either collected in GM-CSF–containing culture media or cytokine-free serum–containing media for 6–12 hours.

### Cell proliferation assay.

We seeded 10,000 cells of various cell lines or primary mouse HSPCs in triplicate in 96-well plates in a graded concentration of cytokines at 100 μL media per well. After 3 days of culture, 3-(4,5-dimethylthiazole-2-yl)-2,5-diphenyl tetrazolium bromide (MTT; Invitrogen) was added to a final concentration of 0.5 mg/mL and incubated for 4 hours at 37°C. 100 μL stopping buffer (5% SDS, 2.5% acetic acid, 50% dimethylformamide) was then added to terminate the reaction. The absorbance was measured on a SpectraMax 190 Microplate Reader at 570 nm.

### Colony-forming cell assay.

For primary human AML cells, 50,000 cells in 1 mL methylcellulose (StemCell) supplemented with 5 U/mL EPO (EPOGEN), 10 ng/mL IL-3, 5 ng/mL SCF and 5ng/mL GM-CSF were thoroughly vortexed, plated in triplicates in 35 mm small petri dishes (Olympus), and kept in an incubator at 37°C and 95% humidity in an atmosphere of 5% CO_2_. The colonies were counted after 10–14 days.

For primary mouse HSPCs, 15,000 cells were resuspended in 1 mL methylcellulose (StemCell) supplemented with 0, 0.2, or 2 ng/mL GM-CSF, thoroughly vortexed, and plated in triplicates in 35 mm small petri dishes (Olympus). The plates were incubated at 37°C and 95% humidity in an atmosphere of 5% CO_2_, and colonies were counted after 7–10 days.

### Bone marrow transplantation.

300,000 LSKs from *Rab27b^fl/fl^* or *Rab27b^fl/fl;vav^* mice infected with retroviruses expressing *NRAS* were mixed with 500,000 Sca1-depleted competitor BM cells from CD45.1^+^ B6.SJL mice, resuspended in 100 μL DPBS (Gibco), and retroorbitally injected into lethally irradiated (a split dose of 10Gy) recipient mice (CD45.1^+^CD45.2^+^ F1). Six to 10 weeks after transplantation, peripheral blood was collected into EDTA-coated tubes (BD Biosciences) for blood counts and flow cytometry for donor cell reconstitution analysis.

### Complete blood count and flow cytometry analysis.

Complete blood count (CBC) analysis of peripheral blood was performed on a Hemavet 950 (Drew Scientific). For donor cell reconstitution analysis, peripheral blood was lysed in RBC lysis buffer (0.8% NH_4_Cl, 10 μM EDTA, pH ~7.4) for 10 minutes at 4°C, then washed with PBS containing 0.5% BSA and 2 mM EDTA buffer. BM and spleen cells were collected and directly subjected to flow cytometric analysis. Cells were incubated with conjugated antibodies (lymphoid panel PE-Cy7-CD45.1, APC-Cy7-CD45.2, PE-B220, and APC-CD3; myeloid panel PE-Cy7-CD45.1, APC-Cy7-CD45.2, PE-Gr1, and APC-Mac1) for 30 minutes at 4°C and resuspended in PBS containing 0.5% BSA and 2mM EDTA buffer plus DAPI, followed by flow cytometry on a BD LSRFortessa Cell Analyzer.

### Statistics.

Volcano plots were generated using Prism 8.0 (GraphPad Software Inc). Colocalization of GFP-NRAS and the PM marker WGA was calculated using Mander’s coefficient on ImageJ. Percentages of cells with PM localized GFP-NRAS were analyzed using Fisher’s exact test. Statistics for qRT-PCR, MTT, CFC, CBC, and flow cytometry assays were performed using unpaired 2-tailed Student’s *t* test, 1-way, or 2-way analysis of variance (Prism 8.0, GraphPad Software Inc). Data are shown as mean ± SD. *P* < 0.05 was considered statistically significant.

### Study approval.

All the animal studies were performed under a protocol approved by the IACUC of the Children’s Hospital of Philadelphia (no. 2021-0781). Deidentified blood or bone marrow samples of human myeloid malignancies were obtained from the Human Stem Cell and Xenograft Core at the University of Pennsylvania under the blanket IRB (no. 703185).

## Author contributions

WT and JGR conceived the project. WT and MRP supervised the studies. WT and JGR designed the experiments and wrote the manuscript. JGR performed the biochemistry and functional assays in cell lines, mouse LSKs, and human primary AML cells. JGR and BX performed animal experiments and flow cytometry. RAO and MRP performed live-cell imaging in SK-MEL-147 cells. KL prepared the samples for SILAC MS. MW performed the ABHD17A protein interaction assay. RW generated TF-1 CBL DKO cells. KMB, AG, and RMO generated *Rab27b ff* and *Rab27b ff;vav* mice. VP reviewed the histology of transplanted mice for diagnosis. KL, RAO, KMB, JZ, EOH, RMO, GMB, and MRP revised the manuscript.

## Supplementary Material

Supplemental data

Supplemental table 1

Supplemental table 2

Supplemental table 3

## Figures and Tables

**Table 1:**
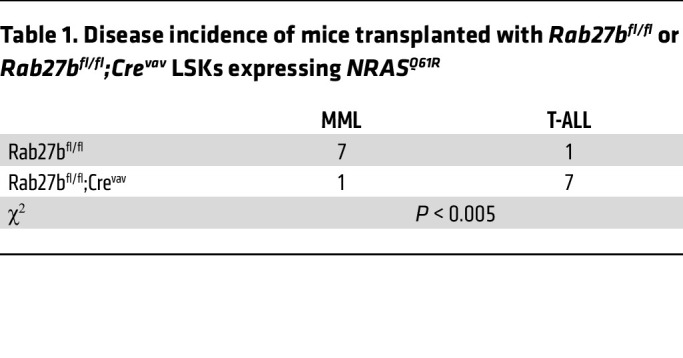
Disease incidence of mice transplanted with *Rab27b^fl/fl^* or *Rab27b^fl/fl^;Cre^vav^* LSKs expressing *NRAS^Q61R^*

**Table 2:**
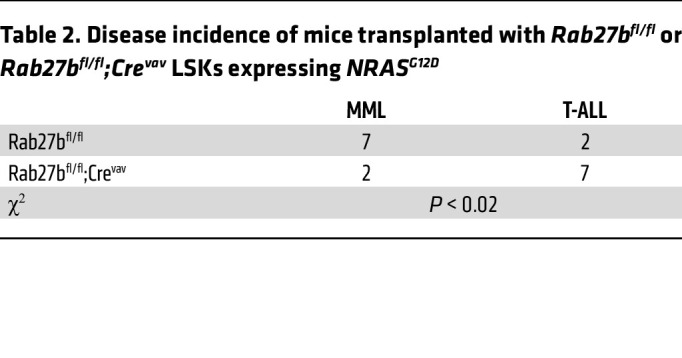
Disease incidence of mice transplanted with *Rab27b^fl/fl^* or *Rab27b^fl/fl^;Cre^vav^* LSKs expressing *NRAS^G12D^*

## Version 1 06/15/2023

Electronic publication
