# Supplementary material for: RAB27B controls palmitoylation-dependent NRAS trafficking and signaling in myeloid leukemia
Source: J Clin Invest. 2023 Jun 15;133(12):e165510. doi: 10.1172/JCI165510 (PMC10266782; doi:10.1172/JCI165510)
Supplement: Supplemental data [file jci-133-165510-s055.pdf]

Supplemental Tables and Figures for

RAB27B Controls Palmitoylation Dependent NRAS Trafficking and Signaling in Leukemia

Jian-gang Ren[1,2,3], Bowen Xing[2,3,#], Rachel A. O'Keefe[4,#], Kaosheng Lv[2,3,5,#], Mengfang Wu[2,3], Ruoxing Wang[2,3], Kaylyn M. Bauer [6], Arevik Ghazaryan[6], George M. Burslem[7], Jing Zhang[8], Ryan M. O'Connell [6], Vinodh Pillai[9], Elizabeth O. Hexner[10], Mark R. Philips[4], and Wei Tong[2,3,*]

Supplemental Table 1. Comparison of total protein levels between CBL/CBL-B double depleted (DKO+D) versus control (Ctrl) TF-1 cells, and between TF-1 cells overexpressing CBLC381A E3-dead mutant versus CBLWT by quantitative mass spectrometry. Related to Fig. 1.

Supplemental Table 2. Clinical and Molecular Characteristics of the MPN samples. Related to Fig. 2.

Supplemental Table 3. Clinical and Molecular Characteristics of the AML samples. Related to Fig. 6.

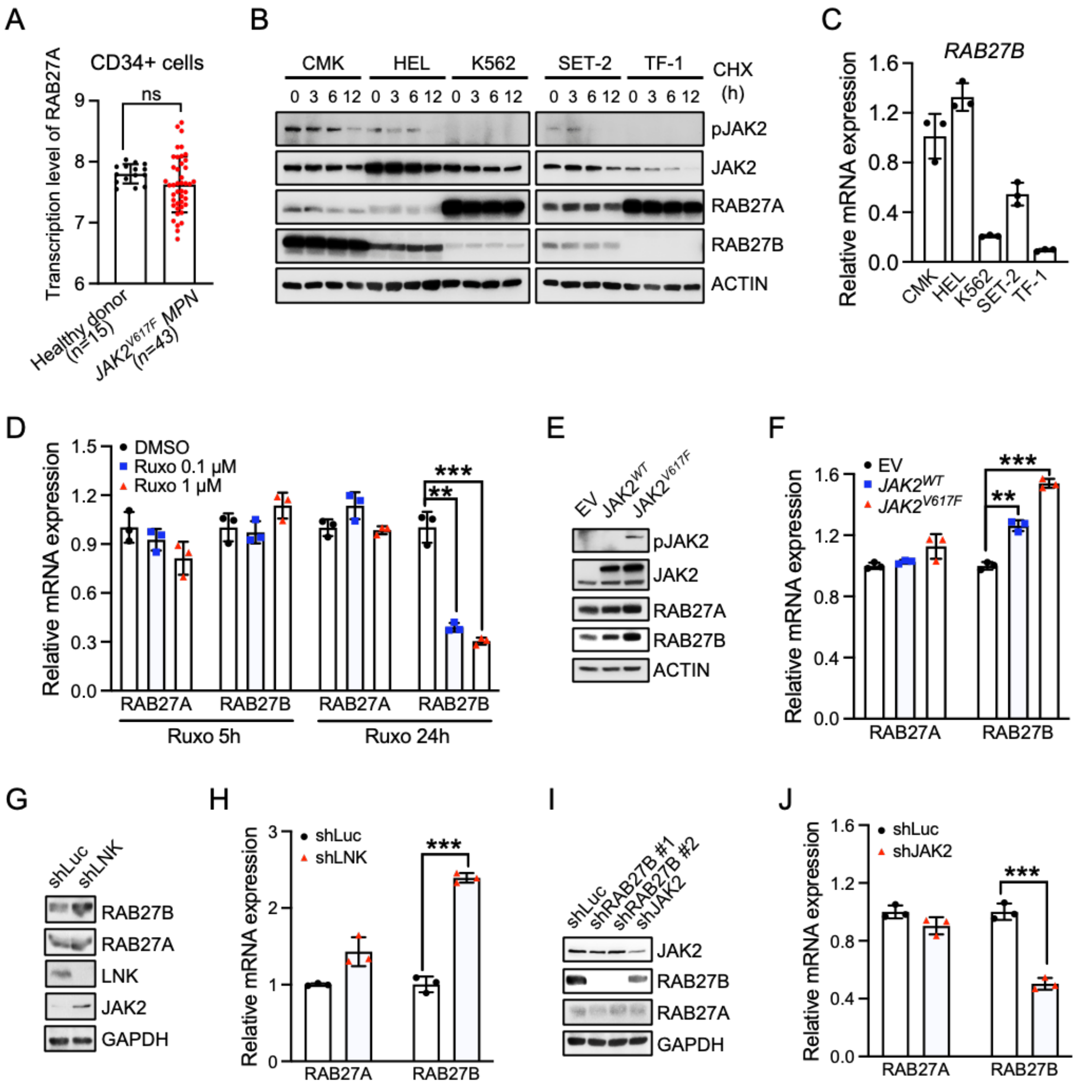

Supplemental Figure 1. Correlation between JAK2 signaling and RAB27 expression.

(A) *RAB27A* mRNA level in bone marrow CD34^{+} cells from healthy donors and patients with $JAK2^{V617F+}$ MPN plotted using the expression data from GSE103176.

(B-C) JAK2 phosphorylation level and RAB27B/A expression in different leukemia cell lines, as determined by WB (A) and qRT-PCR (B).

(D) Relative *RAB27B* and *RAB27A* mRNA level in TF-1 DKO cells after 5h and 24h treatment with 0.1 or 1 μM of the JAK1/2 inhibitor ruxolitinib (Ruxo) in comparison to DMSO-treated control, as determined by qRT-PCR.

(E-F) JAK2 phosphorylation and RAB27B/A expression in TF-1 cells stably expressing empty vector (EV), *WT JAK2*, or $JAK2^{V617F}$ *mutant*, as determined by WB (E) and qRT-PCR (F).

(G-H) RAB27B/A level in *LNK*-deficient TF-1 cells, as determined by WB (G) and qRT-PCR (H).

(I-J) RAB27B/A expression levels in $JAK2^{V617F+}$ HEL cells partially depleted of JAK2 via shRNA-mediated KD (shJAK2) compared to cells treated with shRNA against luciferase (shLuc), as determined by WB (I) and qRT-PCR (J). shRNA against RAB27B was used as an additional control (I).

In all relevant panels, data are represented as mean ± SD. P-values were determined by one-way ANOVA in panels D and F, and by two-tailed Student's *t*-test in panels A, H and J. **, P<0.01; ***, P<0.001; ns: not significant.

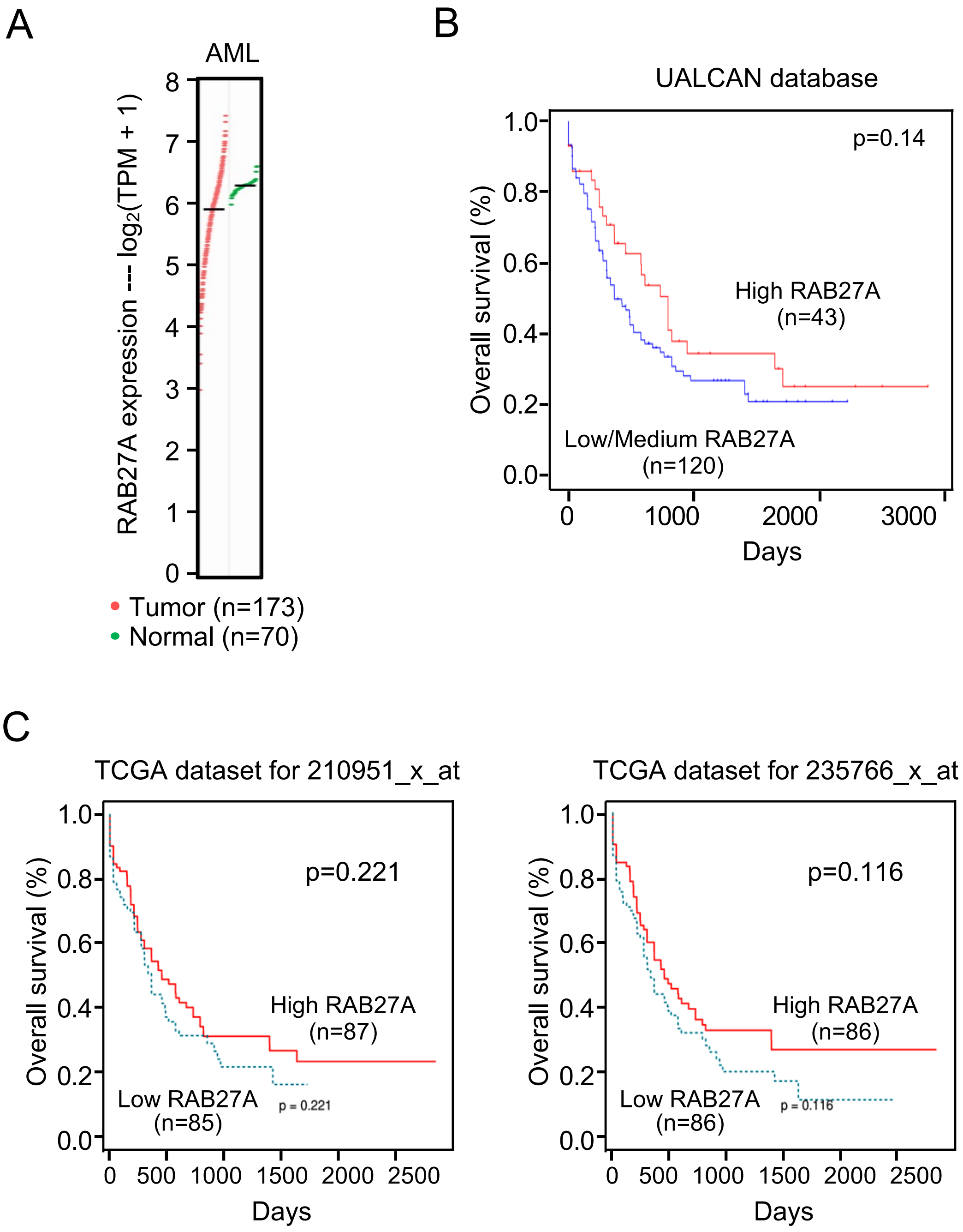

Supplemental Figure 2. ***RAB27A*** **expression level in AML, and examination of its association with AML prognosis.**
(A) *RAB27A* expression levels in AML versus healthy controls (GEPIA Cancer Database).
(B-C) Kaplan-Meier plot of overall survival for AML patients with low or high expression of *RAB27A*. UALCAN (B): top 25% or bottom 75% (low/medium) expression; TCGA database from BloodSpot (C): top 50% or bottom 50% expression. P values determined by log-rank *t*-test are shown.

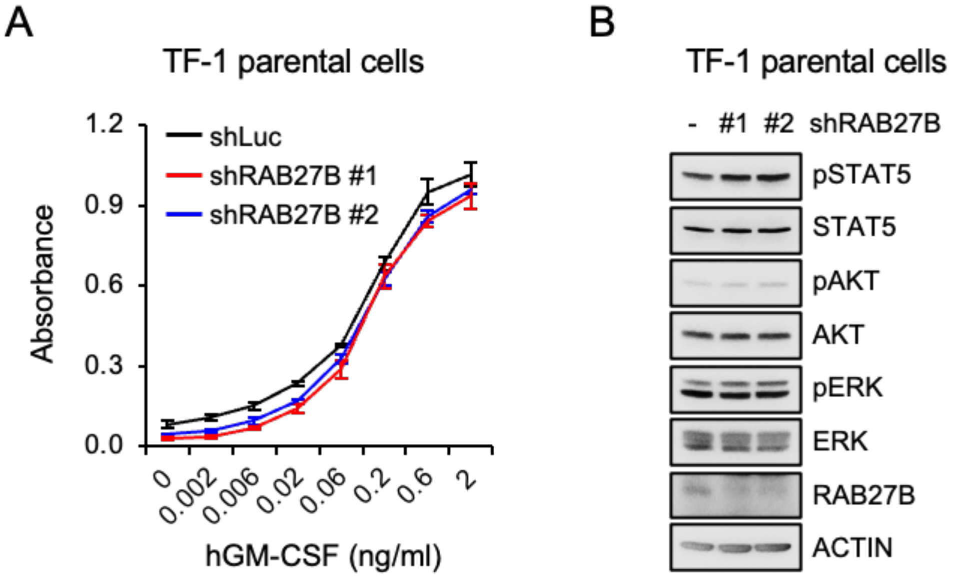

Supplemental Figure 3. *RAB27B* depletion does not affect growth and signaling of parental TF-1 cells.
RAB27B was stably depleted via lentiviral-shRNA mediated KD in TF-1 cells, with shRNA against luciferase (shLuc) as a control.
(A) Cells were cultured in triplicates in different concentrations of human GM-CSF and cell growth after 3 days in culture was determined by MTT absorbance (mean ± SD).
(B) Cell lysates were subjected to WB analysis with the indicated antibodies to examine various signaling pathways.

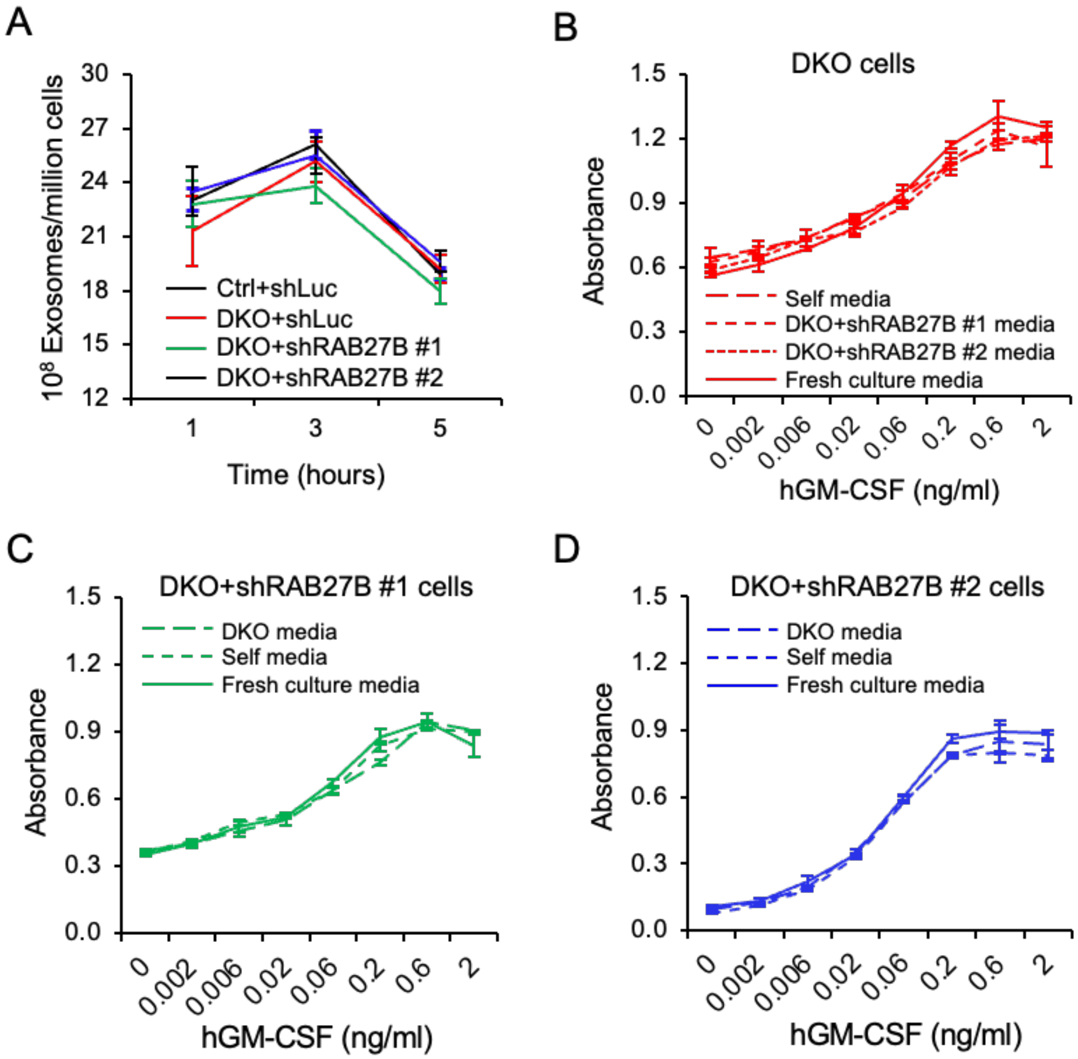

Supplemental Figure 4. ***RAB27B*** **depletion does not affect secretion of exosomes or cytokines in TF-1 DKO cells.**

TF-1 DKO cells were depleted of RAB27B via shRNA-mediated KD, with shRNA against luciferase (shLuc) used as a control.

(A) Quantification of secreted exosomes (mean ± SD) at different time points.

(B-D) TF-1 DKO cells without (B) or with RAB27B knockdown (C, D) were cultured in triplicates in different conditioned media as indicated. Cell growth was determined by MTT absorbance (mean ± SD).

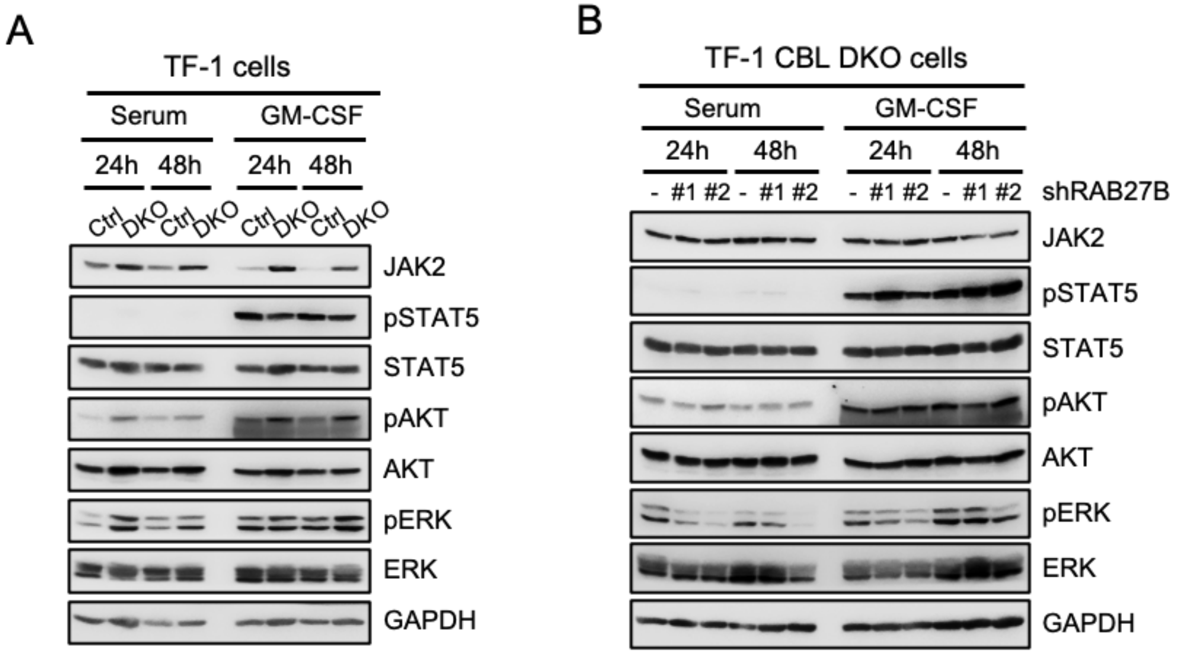

Supplemental Figure 5. ***RAB27B*** **depletion inhibits ERK activation in TF-1 DKO cells.**
(A-B) TF-1 Ctrl versus DKO cells (A) or TF-1 DKO cells with or without RAB27B depletion (B) were cultured in media containing serum only or serum plus GM-CSF. Cells were lysed at the indicated times, and cell lysates were subjected to WB analysis with the indicated antibodies to examine various signaling proteins.

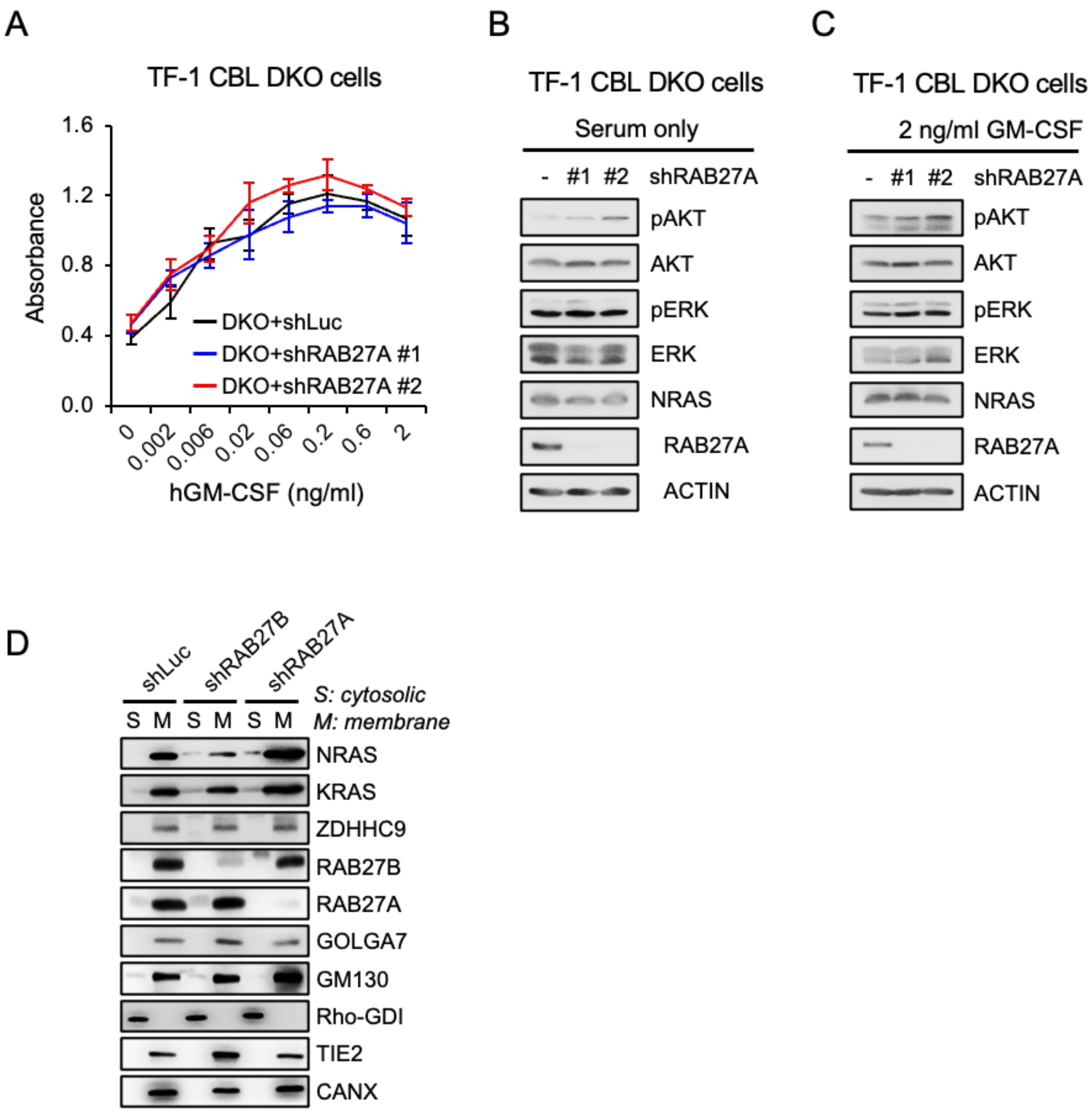

Supplemental Figure 6. *RAB27A* depletion does not affect growth, ERK activation, or NRAS localization of TF-1 DKO cells.

RAB27A was stably depleted via lentiviral-shRNA mediated KD in TF-1 DKO cells, with shRNA against luciferase (shLuc) used as a control.

(A) Cells were cultured in triplicates in different concentrations of GM-CSF and cell growth after 3 days in culture was determined by MTT absorbance (mean ± SD).

(B-C) TF-1 DKO cells were cultured in serum only media (B) or GM-CSF-containing media (C). Cell lysates were subjected to WB analysis with the indicated antibodies.

(D) TF-1 DKO cells with or without depletion of RAB27B or RAB27A were subjected to subcellular fractionation followed by WB analysis with the indicated antibodies.

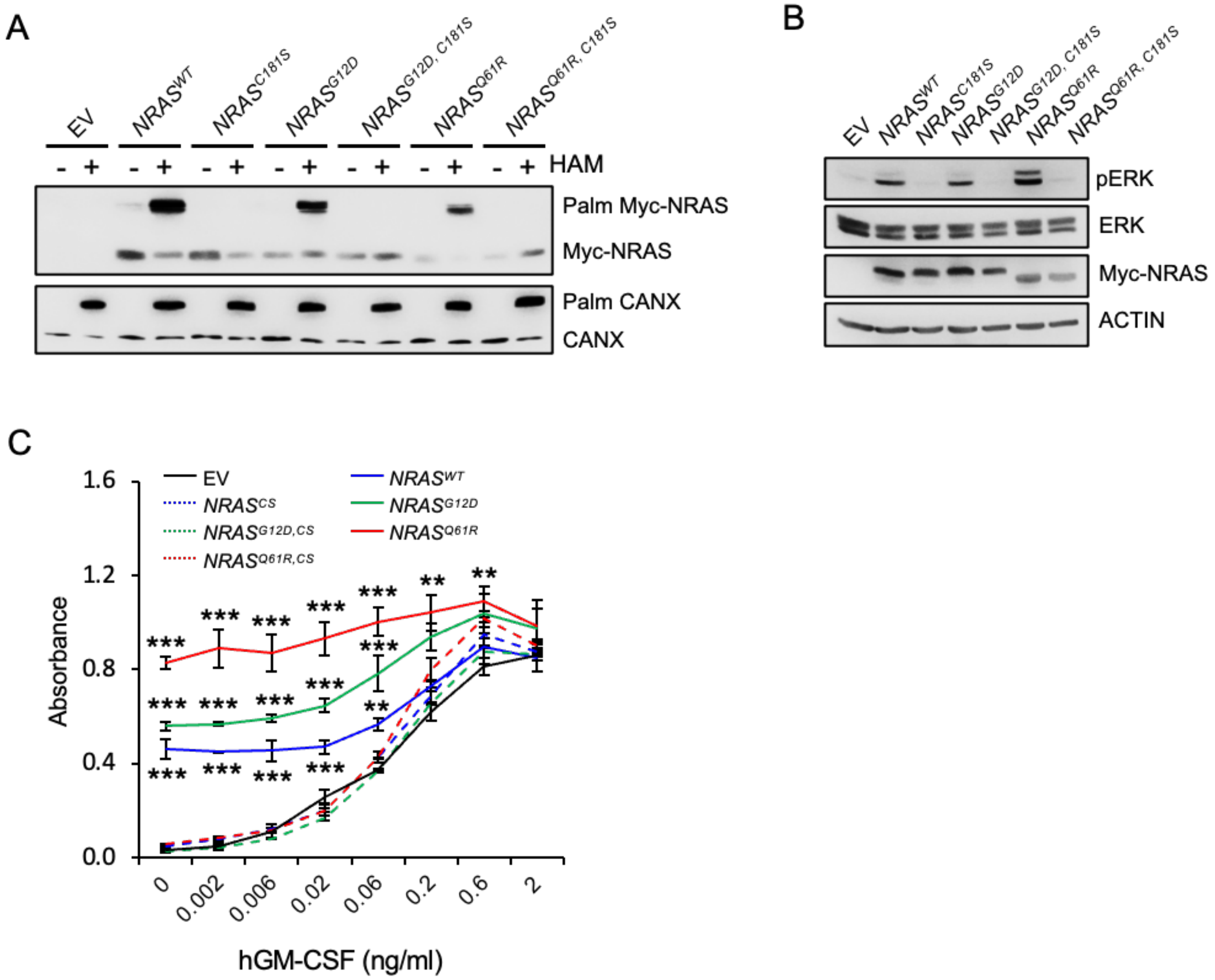

Supplemental Figure 7. Palmitoylation deficiency abrogates oncogenic NRAS-conferred cell growth and ERK activation in TF-1 cells.

TF-1 cells stably expressing WT, G12D or Q61R oncogenic mutant NRAS, or the C181S mutated version of WT or oncogenic mutant NRAS, along with empty vector (EV) control, were established via retroviral infection.

(A-B) Cells were subjected to the APE assay and WB analysis with the indicated antibodies.

(C) Cells were cultured in triplicates in different concentrations of human GM-CSF and cell growth after 3 days in culture was determined by MTT absorbance (mean ± SD).

In all relevant panels, data are represented as mean ± SD, and two-way ANOVA were used, **: $P<0.01$; ***: $P<0.001$, as compared to EV control.

A

B

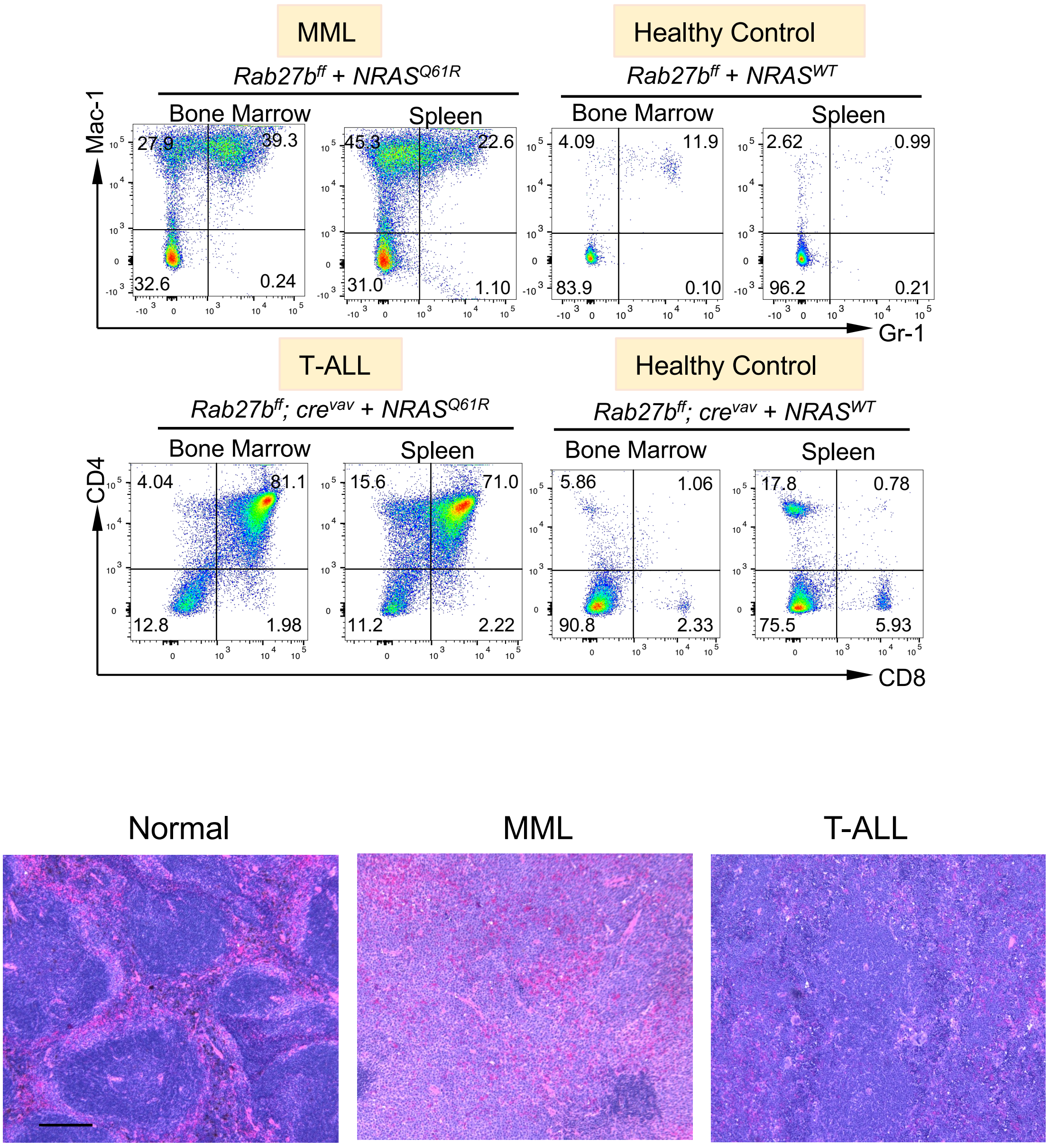

Supplemental Figure 8. *Rab27b* deficiency in mice reduces oncogenic NRAS-mediated myeloid leukemia development in vivo.

(A) Representative flow cytometric plots of bone marrow and spleen of the transplanted mice. GFP+ cells were gated and myeloid (Mac/Gr1) and T cell (CD4/CD8) lineages are shown. The representative flow plots for MML (*Rab27b*$^{f/f}$ + *Nras*^{Q61R}) and for T-ALL (*Rab27b*$^{f/f}$; *cre*^{Vav} + *Nras*^{Q61R}) are shown.

(B) Representative histology (H&E staining) images of the spleens of the mice transplanted with *Nras*^{G12D} are shown. Scale bar indicates 200 µm.

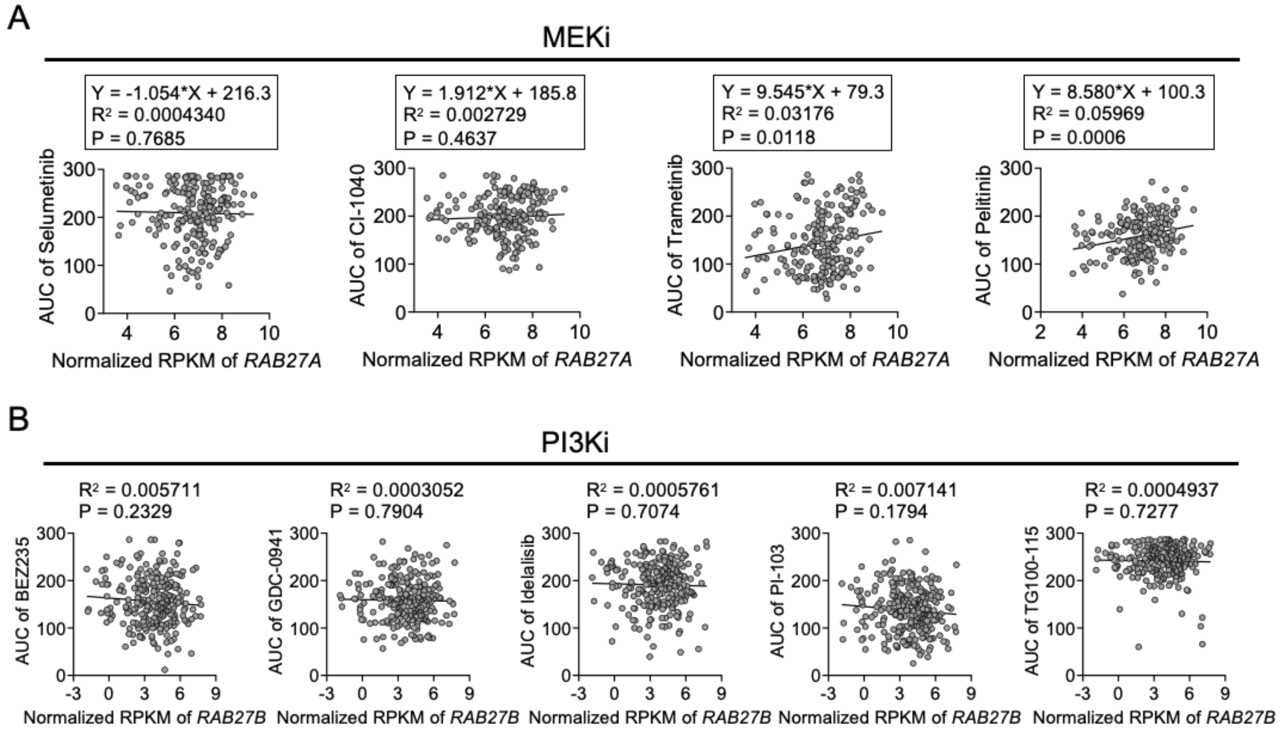

Supplemental Figure 9. No significant correlations between RAB27A expression and sensitivity to MEK inhibitors, or between RAB27B expression and sensitivity to PI3K inhibitors in AML patients.
(A) *RAB27A* expression levels from patients in the BeatAML database are plotted with area under curve (AUC) to different MEK inhibitors. (B) *RAB27B* expression levels from patients in the BeatAML database are plotted with AUC to different PI3K inhibitors. Linear regression trend line, P-value and R-squared value were generated using GraphPad Prism 8.0.

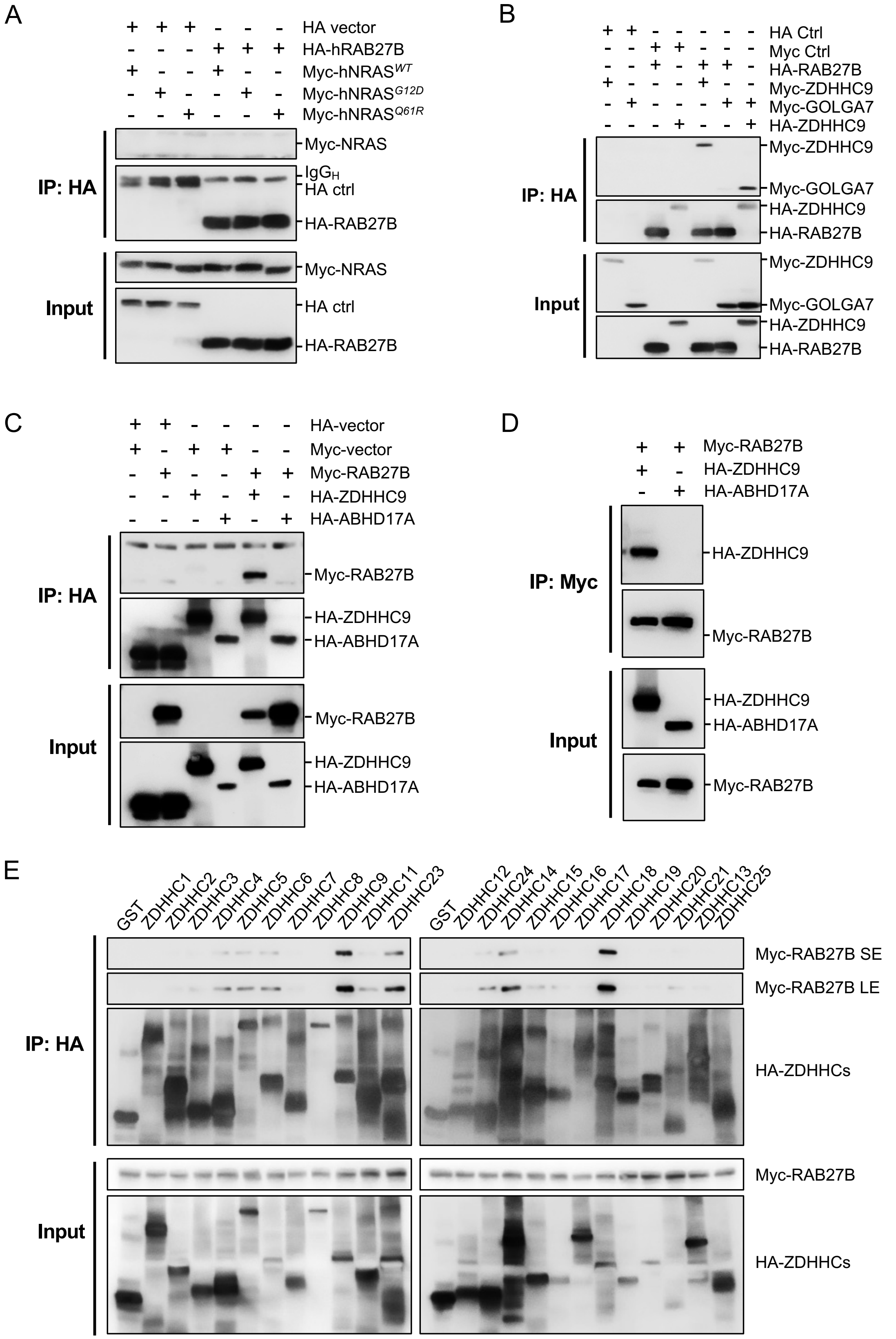

Supplemental Figure 10. Examination of interactions between RAB27B, NRAS, GOLGA7, ABHD17A and ZDHHCs in 293T cells.

293T cells were transfected with constructs to express tagged RAB27B, WT or mutant NRAS, ZDHHCs,

or GOLGA7 as indicated. Cells were then subjected to immunoprecipitation (IP) followed by WB using the indicated antibodies.
(A) Co-IP experiment showing that RAB27B does not interact with WT or oncogenic mutant NRAS.
(B) Co-IP experiment showing that RAB27B binds to ZDHHC9, but not GOLGA7.
(C-D) Co-IP experiment showing that RAB27B binds to ZDHHC9, but not ABHD17A.
(E) Co-IP experiment to examine the interactions between RAB27B and 23 different ZDHHCs.

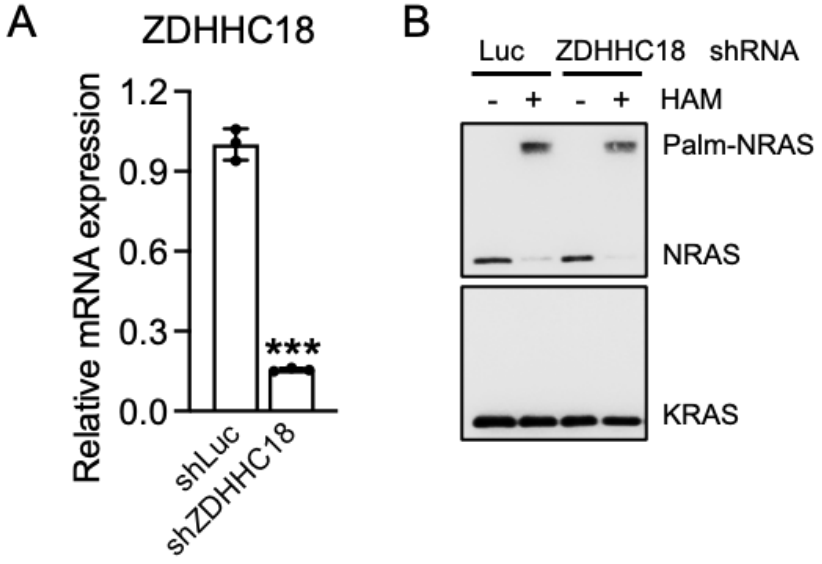

Supplemental Figure 11. ***ZDHHC18*** **depletion does not affect NRAS palmitoylation in TF-1 DKO cells.**

(A) KD efficiency of the *ZDHHC18* shRNA construct in comparison to shRNA against luciferase (shLuc) was examined by qRT-PCR used as a control (mean ± SD). Student's two-tailed *t*-test was used, ***: P<0.001, compared to the shLuc group.

(B) RAS palmitoylation was determined using the APE assay upon *ZDHHC18* depletion in comparison to shLuc.

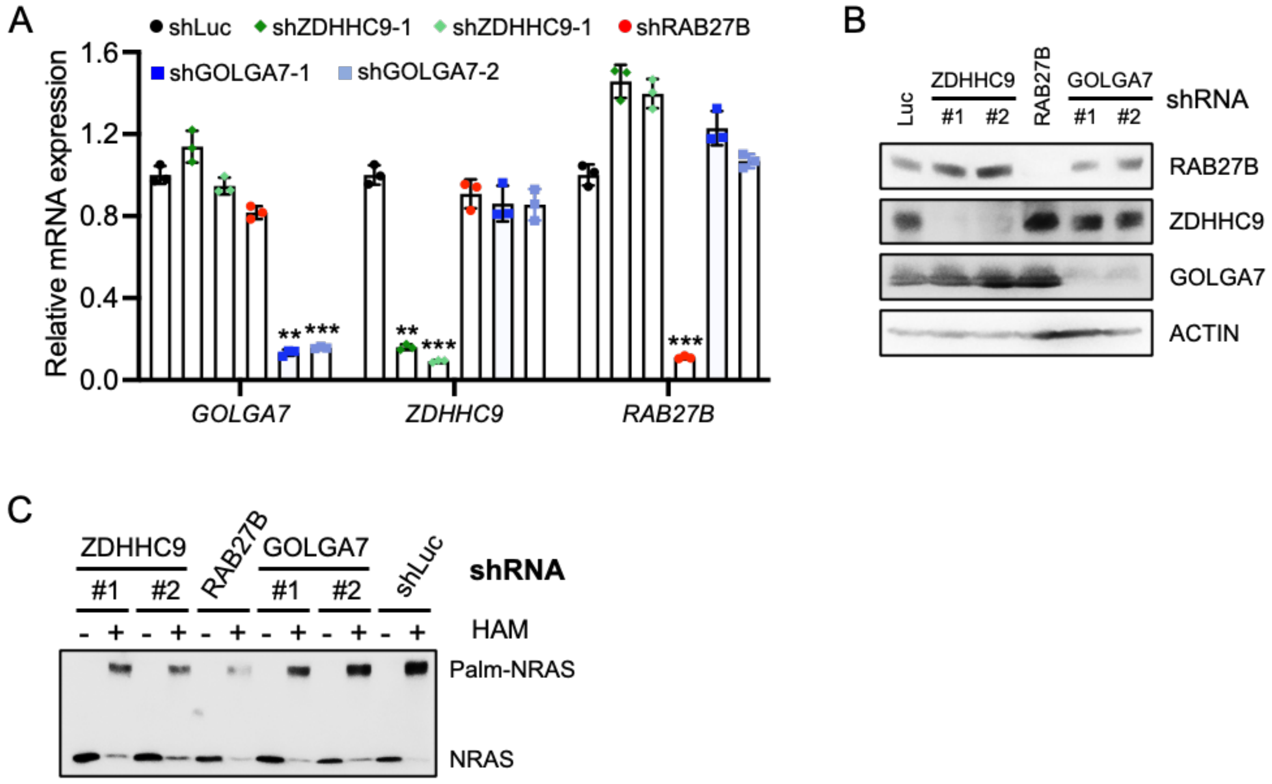

Supplemental Figure 12. Depletion of *ZDHHC9*, *GOLGA7* or *RAB27B* reduces NRAS palmitoylation in TF-1 DKO cells.
(A and B) KD efficiency of ZDHHC9, GOLGA7 and RAB27B shRNA constructs was detected by qRT-PCR (A) and WB (B). Data are represented as mean ± SD. P-values are determined by one-way ANOVA, **, P<0.01; ***, P<0.001 compared to the shLuc group.
(C) *ZDHHC9*, *GOLGA7* or *RAB27B* depletion reduces palmitoylation of endogenous NRAS as determined using the APE assay.
